# Supplementary material for: BioSimulators: a central registry of simulation engines and services for recommending specific tools
Source: Nucleic Acids Res. 2022 May 7;50(W1):W108–14. doi: 10.1093/nar/gkac331 (PMC9252793; doi:10.1093/nar/gkac331)
Supplement: gkac331_Supplemental_File [file gkac331_supplemental_file.pdf]

# BioSimulators: a web-based registry of simulation engines and services for multiscale modeling – Supplementary data

Bilal Shaikh[1], Lucian P. Smith[2], Dan Vasilescu[3], Gnaneswara Marupilla[3], Michael Wilson[3], Eran Agmon[4], Henry Agnew[5], Steven S. Andrews[2], Azraf Anwar[6], Moritz E. Beber[7], Frank T. Bergmann[8], David Brooks[9], Lutz Brusch[10], Laurence Calzone[11], Kiri Choi[12], Joshua Cooper[13], John Detloff[14], Brian Drawert[13], Michel Dumontier[15], G. Bard Ermentrout[16], James R. Faeder[16], Andrew P. Freiburger[17], Fabian Fröhlich[18], Akira Funahashi[19], Alan Garny[9], John H. Gennari[20], Padraig Gleeson[21], Anne Goelzer[22], Zachary Haiman[23], Jan Hasenauer[24], Joseph L. Hellerstein[2], Henning Hermjakob[25], Stefan Hoops[26], Jon C. Ison[27], Diego Jahn[10], Henry V. Jakubowski[28], Ryann Jordan[1], Matúš Kalaš[29], Matthias König[30], Wolfram Liebermeister[22], Rahuman S. Malik Sheriff[25], Synchon Mandal[31], Robert McDougal[32], J. Kyle Medley[33], Pedro Mendes[3], Robert Müller[10], Chris J. Myers[34], Aurelien Naldi[35], Tung V. N. Nguyen[25], David P. Nickerson[9], Brett G. Olivier[36], Drashti Patoliya[37], Loïc Paulevé[38], Linda R. Petzold[39], Ankita Priya[40], Anand K. Rampadarath[9], Johann M. Rohwer[41], Ali S. Saglam[16], Dilawar Singh[42], Ankur Sinha[43], Jacky Snoep[41], Hugh Sorby[9], Ryan Spangler[44], Jörn Starruß[10], Payton J. Thomas[45], David van Niekerk[41], Daniel Weindl[46], Fengkai Zhang[47], Anna Zhukova[48], Arthur P. Goldberg[1], Michael L. Blinov[3], Herbert M. Sauro[2], Ion I. Moraru[3] and Jonathan R. Karr[1,*]

[1]Icahn School of Medicine at Mount Sinai, New York, NY 10029, US, [2]University of Washington, Seattle, WA 98105, US, [3]University of Connecticut School of Medicine, Farmington, CT 06030, US, [4]Stanford University, Stanford, CA 94305, US, [5]LibreTexts, US, [6]New York University, Brooklyn, NY 11201, [7]Unseen Bio ApS, 2100 København Ø, DK, [8]Heidelberg University, 69120 Heidelberg, DE, [9]University of Auckland, 1010 Auckland, NZ, [10]Technical University of Dresden, 01187 Dresden, DE, [11]Institut Curie, 75248 Paris, FR, [12]Korea Institute for Advanced Study, 02455 Seoul, KR, [13]University of North Carolina, Asheville, Ashville, NC 28804, US, [14]Independent, Madison, WI 53705, US, [15]Maastricht University, 6200 Maastricht, NL, [16]University of Pittsburgh, Pittsburgh, PA 15260, US, [17]University of Victoria, Victoria, BC V8P 5C2, CA, [18]Harvard Medical School, Boston, MA 02115 US, [19]Keio University, Yokohama 223-8522, JP, [20]University of Washington, Seattle WA 98019, US, [21]University College London, London WC1E 6BT, UK, [22]Université Paris-Saclay, INRAE, MaIAGE, 78350 Jouy-en-Josas, FR, [23]University of California, San Diego, La Jolla, CA 92093, US, [24]Universität Bonn, 53115 Bonn, DE, [25]European Molecular Biology Laboratory - European Bioinformatics Institute, Hinxton, Cambridge CB10 1SD, UK, [26]University of Virginia, Charlottesville, VA 22904, US, [27]CNRS, UMS 3601, Institut Français de Bioinformatique, IFB-core, 91000 Évry-Courcouronnes, FR, [28]College of Saint Benedict and Saint John's University, St. Joseph, MN 56374, US, [29]University of Bergen, 5020 Bergen, NO, [30]Humboldt University of Berlin, 10115 Berlin, DE, [31]Technical University of Dresden, 01069 Dresden, DE, [32]Yale University, New Haven, CT 06511, US, [33]Autodesk, Inc., San Rafael, CA 94903, US, [34]University of Colorado at Boulder, Boulder CO, 80309, US, [35]Inria Saclay - Île-de-France Research Centre, 91120 Palaiseau, FR, [36]Vrije Universiteit Amsterdam, 1081 HZ Amsterdam, NL, [37]Sarvajanik College of Engineering & Technology, Surat, Gujarat 395001, IN, [38]Centre National de la Recherche Scientifique, 33400 Talence, France, [39]University of California, Santa Barbara, Santa Barbara, CA 93106, US, [40]Birla Institute of Technology, Mesra, Jharkhand 835215, IN, [41]Stellenbosch University, Stellenbosch, 7600, ZA, [42]Subconscious Compute Pvt. Ltd., Bangalore, IN, [43]University College London, London, WC1E 6BT, UK, [44]Allen Institute for Cell Science, Seattle, WA 98109, US, [45]University of Utah, Salt Lake City, UT 84112, US, [46]Helmholtz Zentrum München GmbH and German Research Center for Environmental Health, 85764 Neuherberg, DE, [47]National Institutes of Health, Bethesda, MD 20892, US and [48]Institut Pasteur, 75015 Paris, FR.

*Correspondence: karr@mssm.edu

## Contents


**S1 Biosimulation tools registered with BioSimulators** **3**

**S2 Foundational conventions that enable BioSimulators** **3**

S2.1 Simulation projects: COMBINE archive, SED-ML, and other community resources . . 5
S2.2 Reports of simulation results: Schema for encoding results and metadata into HDF5 10
S2.3 Visualizations of simulation results: PDF and other domain-independent formats . . 11
S2.4 Logs of the execution of simulations: New YAML schema . . . . . . . . . . . . . . . . . 11
S2.5 Simulation tools: Containerized command-line programs . . . . . . . . . . . . . . . . . 12
S2.6 Lower level simulation capabilities: Python APIs . . . . . . . . . . . . . . . . . . . . . 12
S2.7 Capabilities of simulation tools and the results of their verification: New JSON schema 13

**S3 Foundational tools for validating simulation projects and simulation tools** **13**

S3.1 Validation of simulation experiments described with SED-ML . . . . . . . . . . . . . 14
S3.2 Validation of metadata about simulation projects described with RDF-XML . . . . . 14
S3.3 Validation of entire simulation projects . . . . . . . . . . . . . . . . . . . . . . . . . . 14
S3.4 Validation of logs of the execution of simulation projects . . . . . . . . . . . . . . . . 14
S3.5 Validation of the specifications of the capabilities of simulation tools . . . . . . . . . 14
S3.6 Validation of containerized interfaces to simulation tools . . . . . . . . . . . . . . . . 15

**S4 Foundational tools for working BioSimulators' conventions** **16**

S4.1 KiSAO library: Python package for working with KiSAO . . . . . . . . . . . . . . . . 16
S4.2 BioSimulators-utils: Python package for building interfaces to simulators . . . . . . . 17
S4.3 Automated services for recommending simulation algorithms and tools . . . . . . . . 17
S4.4 runBioSimulations: web application for running BioSimulators tools . . . . . . . . . . 18

**S5 Workflow for creating and registering standardized simulation tools** **19**

S5.1 Map the inputs and outputs of the simulation tool to community conventions . . . . 19
S5.2 Encapsulate the tool into a command-line program . . . . . . . . . . . . . . . . . . . 20
S5.3 Encapsulate the command-line interface to the tool into a Docker image . . . . . . . 21
S5.4 Push the Docker image for the tool to a public Docker registry . . . . . . . . . . . . 21
S5.5 Annotate the capabilities of the simulation tool and other metadata . . . . . . . . . . 21
S5.6 Verify that the simulation tool adheres to BioSimulators conventions . . . . . . . . . 22
S5.7 Submit the tool to the BioSimulators registry . . . . . . . . . . . . . . . . . . . . . . . 22
S5.8 Automate the submission of subsequent versions of the tool to BioSimulators . . . . 23

**S6 Architecture, implementation, testing, and deployment of BioSimulators** **23**

S6.1 Architecture . . . . . . . . . . . . . . . . . . . . . . . . . . . . . . . . . . . . . . . . . 23
S6.2 Implementation, testing, and deployment . . . . . . . . . . . . . . . . . . . . . . . . . 23

**S7 Case study: Assessing the reusability of simulation experiments** **25**

**S8 Comparison of BioSimulators with other resources** **27**

**S9 Availability of BioSimulators** **28**

**S10 Community feedback, input, and contributions to BioSimulators** **29**

**S11 Author contributions** **29**

**S12 Funding** **31**

**S13 Acknowledgements** **32**

**Acronyms** **32**

**References** **33**

# S1 Biosimulation tools registered with BioSimulators

As of March 13, 2022, BioSimulators includes 54 simulation tools (Table S1) which support 13 model formats (Table S2), 14 modeling frameworks (Table S3), and 91 simulation algorithms. Importantly, BioSimulators includes standardized interfaces to 21 of these simulation tools (Table S4). Collectively, these 21 standardized interfaces support 11 model formats, 11 modeling frameworks, and 58 simulation algorithms (Table S5). Detailed, up-to-date information about these tools is available at https://biosimulators.org.

# S2 Foundational conventions that enable BioSimulators

To enable software tools to execute simulations consistently, BioSimulators embraces a set of conventions, including formats, ontologies, software tools, and registries, for capturing simulation tools, their inputs (simulation projects) and outputs (reports and visualizations of simulation results), logs of their execution, and specifications of their capabilities (e.g., the simulation algorithms and model formats that they support) (Figure S1). As much as possible, BioSimulators embraces existing domain and domain-independent conventions. In particular, BioSimulators embraces several existing domain formats for capturing the inputs to simulation tools (e.g., model formats such as CellML (54)), and BioSimulators leverages several existing domain-independent formats for capturing simulation tools and their outputs (e.g., Docker and HDF5 (104)). Where necessary, we have refined these domain formats and customized these domain-independent formats.

First, BioSimulators uses Docker to encapsulate simulation tools and their dependencies into portable images that provide consistent command-line programs. The inputs to these containerized command-line programs are COMBINE archives (105) that use Simulation Experiment Description Markup Language (SED-ML; 106–108) files to describe simulation experiments involving models described in model formats such as BNGL, CellML, or SBML and simulation algorithms described using the Kinetic Simulation Algorithm Ontology (KiSAO; 109, 110). The outputs of these containerized command-line programs are Hierarchical Data Format 5 (HDF5) (104) and Portable Document Format (PDF) files that capture reports and plots of simulation results that are specified in the input SED-ML files and Yet Another Markup Language (YAML) files that capture logs of their execution, such as the algorithm that the simulator used to execute each simulation and its console output. The HDF5 files of simulation results facilitate further visualization and analysis using tools such as Vega (111). When simulations do not succeed as intended, the YAML logs can provide valuable feedback for improvement. Both to help investigators develop these command-line programs and to enable consistent lower-level access to simulation capabilities, BioSimulators also defines a convention for Python APIs for simulation tools and provides a template for containerizing such APIs. To help investigators find tools that can execute specific types of models, we created a JSON schema for capturing the capabilities of simulation tools. This format uses the thE Data And Methods ontology (EDAM; 112, 113), the KiSAO ontology, the Systems Biology Ontology (SBO; 110), and the Semanticscience Integrated Ontology (SIO; 114) to capture the model formats, modeling frameworks, and simulation algorithms that each simulation tool supports.

This section summarizes these formats and ontologies. Detailed information about these formats and examples is available at https://docs.biosimulations.org.

**Table S1: Overview of the specifications of the simulation tools registered with BioSimulators as of March 13, 2022.** Detailed, up-to-date information is available at https://biosimulators.org.

| Name | Ref | Model formats | Model. frameworks | Sim. algorithms | Std. interface |
|---|---|---|---|---|---|
| AMICI | 1 | SBML | g | 2 | ✓ |
| BioNetGen | 2 | BNGL, SBML | g, h, i | 4 | ✓ |
| BioUML | 3 | SBML | a, g, h | 9 | |
| BoolNet | 4 | SBML | f | 3 | ✓ |
| boolSim | 5 | SBML | f | 2 | |
| Brian 2 | 6 | NeuroML/LEMS | g | 3 | ✓ |
| CBMPy | 7 | SBML | a | 4 | ✓ |
| CellNetAnalyzer | 8 | SBML | a, f | 5 | |
| COBRA Toolbox | 9 | SBML | a, c, d | 12 | |
| COBRApy | 10 | SBML | a | 4 | ✓ |
| COPASI | 11 | SBML | b, g, h, n | 10 | ✓ |
| E-Cell 4 | 12 | SBML | h, m | 4 | |
| EpiLog | 13 | SBML | f | | |
| Escher-FBA | 14 | SBML | a | 1 | |
| FlexFlux | 15 | SBML | a, c | 3 | |
| Fluxer | 16 | SBML | a | 1 | |
| Genetic Network Analyzer | 17 | SBML | f | | |
| genYsis | 18 | SBML | f | 2 | |
| GillesPy2 | 19 | SBML | g, h | 8 | ✓ |
| GINsim | 20 | SBML, ZGINML | f | 7 | ✓ |
| iBioSim | 21 | SBML | a, b, g, h | 14 | |
| jNeuroML | 22 | NeuroML/LEMS | g | 2 | |
| JSim | 23 | CellML, SBML | g, l | 11 | |
| JWS Online | 24 | SBML | a, g | 2 | |
| Kappa | 25 | Kappa | i | 1 | |
| Lattice Microbes | 26 | | m | 1 | |
| LibSBMLSim | 27 | SBML | g | 9 | ✓ |
| MaBoSS | 28 | SBML | f | 1 | |
| MASSpy | 29 | SBML, SBML | g | 4 | ✓ |
| MCell | 30 | SBML | j | | |
| MetaNetX | 31 | SBML | a | 2 | |
| mlxM | 32 | pharmML | g | | |
| mlxR | 32 | pharmML | g | | |
| Morpheus | 33 | MorpheusML, SBML | g, h, l, m, n | 11 | |
| NetPyNe | 34 | NeuroML/LEMS, HOC | g | 1 | ✓ |
| NEURON | 35 | NeuroML/LEMS, HOC | g | 1 | ✓ |
| Open Knee | 36 | | l | 1 | |
| OpenCOR | 37 | CellML | g | 6 | ✓ |
| OpenSim | 38 | | | | |
| OptFlux | 39 | SBML | a | 5 | |
| pyNeuroML | 22 | NeuroML/LEMS | g | 2 | ✓ |
| PySB | 40 | BNGL,Kappa, SBML | g, h, i | 11 | |
| PySCeS | 41 | SBML | g | 2 | ✓ |
| RAVEN | 42 | SBML | a | 2 | |
| RBApy | 43 | RBA XML | k | 1 | ✓ |
| SBSCL | 44 | SBML | a, g, h, n | 14 | |
| Simmune | 45 | SBML | i | | |
| SimVascular | 46 | | m | | |
| Smoldyn | 47 | Smoldyn | j | 1 | ✓ |
| Tellurium | 48 | SBML | g, h | 6 | ✓ |
| The Cell Collective | 49 | SBML, SBML | a, f | 6 | |
| Virtual Cell (VCell) | 50 | SBML, VCML | e, g, h, i, l | 14 | ✓ |
| winBEST-KIT | 51 | SBML | g | | |
| XPP | 52 | XPP ODE | g, h | 15 | ✓ |

**Table S2: Overview of the model formats specified for the interfaces to simulation tools registered with BioSimulators as of March 13, 2022.** Detailed, up-to-date information is available at https://biosimulators.org.

| Name | Acronym | Refs | Modeling frameworks | Simulation algorithms | Simulation tools |
|---|---|---|---|---|---|
| BioNetGen Language | BNGL | 53 | 3 | 10 | 2 |
| CellML | | 54 | 3 | 25 | 3 |
| GINsim Markup Language | ZGINML | 20 | 1 | 7 | 1 |
| High Order Calculator | HOC | 35 | 1 | 1 | 2 |
| Kappa language | | 25 | 1 | 1 | 2 |
| Morpheus Markup Language | MorpheusML | 33 | 5 | 11 | 1 |
| NeuroML/Low Entropy Model Specification | NeuroML/LEMS | 22, 55 | 1 | 4 | 5 |
| Resource Balance Analysis XML Format | RBA XML | 43 | 1 | 1 | 1 |
| Smoldyn simulation language | | 47 | 1 | 1 | 1 |
| Systems Biology Markup Language | SBML | 56 | 13 | 84 | 31 |
| Flux Balance Constraints Package | SBML-fbc | 57 | 3 | 15 | 13 |
| Hierarchical Model Composition Package | SBML-comp | 58 | 3 | 3 | 1 |
| Multistate, Multicomponent and Multicompartment Species Package | SBML-multi | 59 | 2 | 4 | 2 |
| Qualitative Models Package | SBML-qual | 60 | 2 | 8 | 6 |
| VCell Markup Language | VCML | 50 | 4 | 14 | 1 |
| XPP ODE format | | 52 | 2 | 15 | 1 |

**Table S3: Overview of the modeling frameworks specified for the interfaces to simulation tools registered with BioSimulators as of March 13, 2022.** Table S1 uses the values in the 'Label' column to indicate the frameworks supported by each simulation tool. Detailed, up-to-date information is available at https://biosimulators.org.

| Label | Name | Model formats | Simulation algorithms | Simulation tools |
|---|---|---|---|---|
| a | Flux balance | 1 | 12 | 13 |
| b | Hybrid deterministic continuous-discrete non-spatial | 1 | 3 | 1 |
| c | Hybrid flux balance-deterministic continuous non-spatial | 1 | 2 | 3 |
| d | Hybrid flux balance-logical-deterministic continuous non-spatial | 1 | 1 | 1 |
| e | Hybrid stochastic continuous-discrete non-spatial | 2 | 3 | 1 |
| f | Logical | 2 | 7 | 5 |
| g | Non-spatial continuous | 9 | 36 | 23 |
| h | Non-spatial discrete | 4 | 14 | 11 |
| i | Particle-based discrete non-spatial | 3 | 2 | 3 |
| j | Particle-based discrete spatial | 3 | 1 | 2 |
| k | Resource balance | 1 | 1 | 1 |
| l | Spatial continuous | 3 | 5 | 2 |
| m | Spatial discrete | 1 | 3 | 1 |
| n | Stochastic non-spatial continuous | 2 | 2 | 2 |

## S2.1 Simulation projects: COMBINE archive, OMEX, SED-ML, KiSAO, EDAM, and other formats and ontologies

BioSimulators embraces existing community standards for describing simulations. The primary inputs to BioSimulators simulation tools are COMBINE archives that contain one or more SED-ML files that describe one or more simulation experiments of one or more models and Open Modeling

**Table S4: Overview of the standardized interfaces to simulation tools registered with BioSimulators as of March 13, 2022.** The 'Modeling framework' and 'Model format' columns indicate the modeling frameworks and model formats that each tool supports. Detailed, up-to-date information is available at https://biosimulators.org.

| | | Modeling framework | | | | | | | | | | | Modeling format | | | | | | | | |
|---|---|---|---|---|---|---|---|---|---|---|---|---|---|---|---|---|---|---|---|---|---|
| Simulator | Ref. | Flux balance | Hybrid det cont-disc non-spatial | Hybrid stochastic cont-disc non-spatial | Logical | Non-spatial continuous | Non-spatial discrete | Particle-based disc non-spatial | Particle-based disc spatial | Resource balance | Spatial continuous | Stochastic non-spatial continuous | BNGL(53) | CellML (54) | ZGINML (20) | NeuroML/LEMS (22) | RBA XML (43) | SBML (56) | Smoldyn (47) | VCML (50) | XPP ODE (52) |
| AMICI | 1 | | | | | ✓ | | | | | | | | | | | | ✓ | | | |
| BioNetGen | 53 | | | | | ✓ | ✓ | ✓ | | | | | ✓ | | | | | | | | |
| BoolNet | 4 | | | | ✓ | | | | | | | | | | | | | ✓ | | | |
| Brian 2 | 6 | | | | | ✓ | | | | | | | | | | ✓ | | | | | |
| CBMPy | 7 | ✓ | | | | | | | | | | | | | | | | ✓ | | | |
| COBRApy | 10 | ✓ | | | | | | | | | | | | | | | | ✓ | | | |
| COPASI | 11 | | ✓ | | | ✓ | ✓ | | | | | ✓ | | | | | | ✓ | | | |
| GillesPy2 | 19 | | | | | ✓ | ✓ | | | | | | | | | | | ✓ | | | |
| GINsim | 20 | | | | ✓ | | | | | | | | | | ✓ | | | ✓ | | | |
| LibSBMLSim | 27 | | | | | ✓ | | | | | | | | | | | | ✓ | | | |
| MASSpy | 29 | | | | | ✓ | | | | | | | | | | | | ✓ | | | |
| NetPyNe | 34 | | | | | ✓ | | | | | | | | | | ✓ | | | | | |
| NEURON | 35 | | | | | ✓ | | | | | | | | | | ✓ | | | | | |
| OpenCOR | 37 | | | | | ✓ | | | | | | | | ✓ | | | | | | | |
| pyNeuroML | 22 | | | | | ✓ | | | | | | | | | | ✓ | | | | | |
| PySceS | 41 | | | | | ✓ | | | | | | | | | | | | ✓ | | | |
| RBApy | 43 | | | | | | | | | ✓ | | | | | | | ✓ | | | | |
| Smoldyn | 47 | | | | | | | | ✓ | | | | | | | | | | ✓ | | |
| Tellurium | 48 | | | | | ✓ | ✓ | | | | | | | | | | | ✓ | | | |
| VCell | 50 | | | ✓ | | ✓ | | | ✓ | | ✓ | | | | | | | ✓ | | ✓ | |
| XPP | 52 | | | | | ✓ | ✓ | | | | | | | | | | | | | | ✓ |

EXchange (OMEX; 105) files that capture metadata about the COMBINE archive, such as the organisms, pathways, reactions, proteins, and genes which the simulations in the archive represent; journal articles which reported the simulations in the archive; and the authors of the archive. These COMBINE archives and SED-ML files rely on SED-ML URNs and URIs for model formats such as BNGL, CellML, and SBML to describe the models involved in each simulation and ids for **KiSAO** concepts to describe the algorithms that should be executed for each task and their parameters.

To achieve our goal of making it easier for investigators to execute simulations, these conventions must support a broad range of simulations, and the conventions must be used consistently across the community. Toward that end, we significantly refined and expanded SED-ML, KiSAO, and

**Table S5: Overview of the simulation algorithms supported by the standardized interfaces to simulation tools registered with BioSimulators as of March 13, 2022.** Detailed, up-to-date information is available at https://biosimulators.org.

| Simulation algorithm | | Model formats | Simulation tools |
|---|---|---|---|
| Adams-Bashforth method | 61 | 2 | 2 |
| Adams-Moulton method | 62 | 2 | 2 |
| Adaptive explicit-implicit tau-leaping method | 63 | 1 | 1 |
| Asynchronous logical model simulation method | 64 | 2 | 2 |
| Backward differentiation formula | 65 | 2 | 2 |
| BDD logical model trap space identification method | | 2 | 1 |
| Brownian diffusion Smoluchowski method | 66 | 3 | 2 |
| Cash-Karp method | 67 | 1 | 1 |
| Crank-Nicolson method | 68 | 1 | 1 |
| CVODE | 69 | 8 | 9 |
| CVODES | 70 | 1 | 1 |
| Dormand-Prince 8(5,3) method | 71 | 2 | 2 |
| Dormand-Prince method | 71 | 2 | 2 |
| Euler backward method | 72 | 2 | 2 |
| Euler forward method | 61 | 6 | 8 |
| Explicit fourth-order Runge-Kutta method | 73 | 6 | 8 |
| Fehlberg method | 74 | 2 | 4 |
| Flux balance analysis (FBA) | 75 | 1 | 2 |
| Flux variability analysis (FVA) | 76 | 1 | 2 |
| Fully-implicit regular grid finite volume method | 77 | 2 | 1 |
| Geometric flux balance analysis | 78 | 1 | 1 |
| Gibson-Bruck next reaction algorithm | 79 | 2 | 2 |
| Gillespie direct algorithm | 80 | 3 | 5 |
| Heun method | 61 | 2 | 2 |
| Hybrid adaptive Gibson-Milstein method | 81 | 2 | 1 |
| Hybrid Gibson-Bruck Next Reaction method/LSODA method | 82, 83 | 1 | 1 |
| Hybrid Gibson-Bruck Next Reaction method/RK-45 method | 82, 83 | 1 | 1 |
| Hybrid Gibson-Bruck Next Reaction method/Runge-Kutta method | 82, 83 | 1 | 1 |
| Hybrid Gibson-Euler-Maruyama method | 81 | 2 | 1 |
| Hybrid Gibson-Milstein method | 81 | 2 | 1 |
| Hybrid tau-leaping method | 19 | 1 | 1 |
| IDA | 84 | 2 | 2 |
| KINSOL | 84 | 1 | 1 |
| Klarner ASP logical model trap space identification method | 85 | 2 | 1 |
| LSODA | 86 | 1 | 2 |
| LSODA/LSODAR hybrid method | 11 | 1 | 1 |
| Midpoint method | 87 | 5 | 3 |
| Naldi MDD logical model stable state search method | 88 | 2 | 1 |
| NFSim agent-based simulation method | 89 | 1 | 1 |
| NLEQ2 | 90, 91 | 1 | 1 |
| Numerical Recipes in C "quality-controlled Runge-Kutta" method | 92 | 1 | 1 |
| Numerical Recipes in C "stiff" Rosenbrock method | 92 | 1 | 1 |
| Parsimonious enzyme usage FBA (minimum sum of absolute fluxes) | 93 | 1 | 2 |
| Parsimonius FBA (minimum number of active fluxes) | 94 | 1 | 1 |
| Partitioned leaping method | 95 | 1 | 1 |
| Partitioned Runge-Kutta method | 96 | 1 | 1 |
| Probabilistic logical model simulation method | 64 | 2 | 2 |
| Radau method | 97 | 1 | 1 |
| Resource Balance Analysis | 98 | 1 | 1 |
| Rosenbrock method | 99 | 1 | 1 |
| Second order backward implicit product Euler scheme | | 1 | 1 |
| Semi-implicit regular grid finite volume method | 77 | 2 | 1 |
| Sequential logical simulation method | | 2 | 1 |
| Stochastic second order Runge-Kutta method | 100, 101 | 1 | 1 |
| Synchronous logical model simulation method | 64 | 2 | 2 |
| tau-leaping method | 102 | 1 | 2 |
| VODE | 103 | 1 | 1 |
| ZVODE | 103 | 1 | 1 |

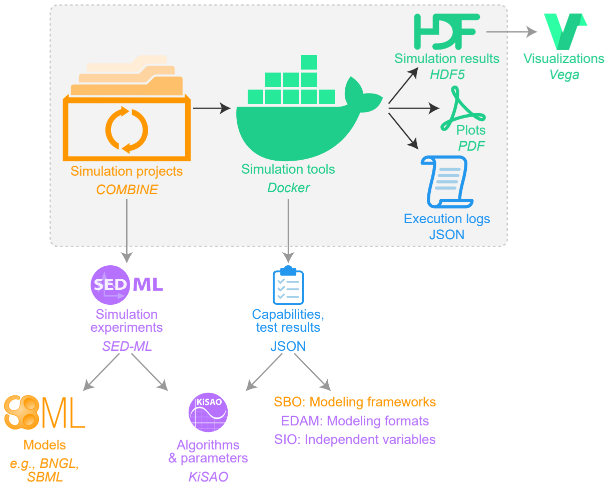


**Figure S1: BioSimulators standardizes the simulation of a broad range of models by leveraging several new and existing formats and ontologies to encapsulate the details of individual model formats, modeling frameworks, and simulation algorithms.** Specifically, BioSimulators standardizes simulation tools as Docker images whose primary inputs are COMBINE archives that contain descriptions of simulation of experiments and whose primary outputs are reports and plots of the results of these simulation experiments in HDF5 and PDF formats and metadata about their execution in BioSimulators' YAML log format. To help investigators find tools that can execute specific types of models, BioSimulators uses a new JSON schema to describe their capabilities. The grey box indicates the primary inputs and outputs of BioSimulators-standardized simulation tools. Orange and purple indicate existing modeling formats and ontologies that BioSimulators embraces for the inputs to simulation tools; orange indicates resources that BioSimulators embraces without modification; purple indicates resources that we refined or expanded to enable BioSimulators. Green indicates existing domain-independent formats and resources that we customized for simulation tools and their outputs. Blue indicates new resources that we developed to enable BioSimulators.

EDAM as outlined below.

#### S2.1.1 Refinement of the SED-ML format for simulation experiments

To enable us to use SED-ML to consistently execute a broad range of simulation experiments with a broad range of model formats, simulation algorithms, and simulation tools, we worked with the community to significantly clarify, enhance, expand, and formalize SED-ML. This resulted in a new version of SED-ML (L1V4), which was released last year (108).

These enhancements included expanding SED-ML's registry of model formats, generalizing the targets of model changes and simulation observables to support non-XML languages and XML languages that use conventions other than XML XPaths to address parameters and observables, and introducing a new class for capturing analyses of models beyond the calculation of steady states and simulation of uniform time courses such as simulations of adaptive time courses and calculations of attractors.

To make it easier for investigators to exchange simulation experiments between simulation tools and enable modular execution of simulation experiments by collections of tools, we revised the specifications of SED-ML to be more concrete. For example, this included clarifying how XML XPaths for model changes and simulation observables should be interpreted and detailing how repeated tasks should be interpreted.

In particular, we added rules for SED-ML documents to the specifications of SED-ML. Each rule includes a unique code that software tools that validate SED-ML documents can use to direct users to further information about particular issues. The majority these rules formalize existing conventions for using SED-ML. For example, this includes rules that ensure that each model can be resolved to a specific URI and that repeated tasks can be resolved to a set of concrete tasks. As described below, we developed software to validate SED-ML documents according to these rules. This software enables investigators to quickly find errors in SED-ML documents. This software is available as a web application, REST API, command-line program, and Python API. This software is also embedded into most of the BioSimulators interfaces to simulation tools. Furthermore, we revised the canonical SED-ML examples to adhere to these rules.

To ensure that BioSimulators interfaces to simulation tools interpret SED-ML consistently, we developed more concrete guidelines for using SED-ML. For example, these guidelines outline how each model language should be used in conjunction with SED-ML, how the values of non-scalar algorithm parameters should be encoded into SED-ML, how reports should be encoded in HDF5, and where outputs should be exported. These guidelines are available at https://docs.biosimulations.org.

Currently, the majority of BioSimulators interfaces to simulation tools support (a) the subset of L1V4 that was part of L1V3, with the clarifications, enhancements, and formalization introduced in L1V4 and (b) styles for plots which were introduced in L1V4. Going forward, we plan to encourage simulation software developers to fully support L1V4. This will include advancing BioSimulators-utils to support L1V4.

#### S2.1.2 Refinement and expansion of the KiSAO ontology of simulation methods

To enable investigators to find simulation tools with the specific capabilities required to execute particular simulation projects, we significantly refined and expanded the KiSAO ontology. First, we added 150 (28%) more concepts to capture the algorithms and algorithm parameters registered with BioSimulators. Second, we refined the parent-child and other relationships between the simulation algorithms to enable us to query the ontology for groups of similar algorithms, such as pairs of mathematically-equivalent algorithms (e.g., Gillespie's Direct Method (80) and the Gibson-Bruck Next Reaction Method (79)), pairs of algorithms that closely approximate the same mathematics (e.g., Gillespie's Direct Method and the tau-leaping method (102)), pairs of algorithms that make dissimilar approximations to the same mathematics (e.g., tau-leaping method and CVODE (69)), and pairs of algorithms that predict similar variables despite solving different mathematical problems (e.g., FBA and pFBA). Third, we added 39 (7%) more concepts to capture the outputs of simulation methods, such as fluxes and concentrations.

#### S2.1.3 Expansion of the EDAM ontology of formats and SED-ML registry of model formats

To enable consistent descriptions of the model formats involved in COMBINE archives, the model formats which particular simulation tools can execute, and the formats of the outputs of simulations, we have proposed 53 additional concepts. To enable the modeling community to better utilize EDAM, we also filled gaps in the annotation of several existing concepts for modeling formats, such as annotating additional media types and file extensions and refining the categorization of modeling formats. We similarly expanded the SED-ML registry of model formats to enable SED-ML to

describe simulations involving a broader range of model formats, such as BNGL and the XPP ODE format.

#### S2.1.4 Expansion of the SIO ontology of physical, processual, and informational entities

To enable consistent descriptions of the dimensions of the outputs of simulations, we also added a few concepts to the SIO ontology, such as to describe a dimension that represents instances of an ensemble of runs of a stochastic simulation.

### S2.2 Reports of simulation results: Schema for encoding results and metadata into HDF5

The primary outputs of simulation tools are data sets of the predictions of the steady-states, dynamical trajectories, or other properties of biological systems. These data sets are typically represented as tables, or more generally, as multidimensional matrices. For example, dynamical trajectories of the concentrations of proteins predicted by a continuous kinetic simulation could be represented as a two-dimensional table whose columns represent individual proteins and whose rows represent the time points of the predicted trajectories of the concentrations of those proteins. An ensemble of dynamical trajectories of the counts of RNAs predicted by a set of 3-dimensional Monte Carlo simulations could be represented as a five-dimensional matrix whose dimensions represent the mRNA species; the time points of the predicted trajectories; and the x, y, and z coordinates of the simulated subvolumes. Reaction fluxes predicted by a FBA simulation could be represented as a one-dimensional vector whose entries represent the predicted flux of each reaction.

Because the predictions of simulations are the starting point for visualizations and other analyses, investigators need to be able to obtain simulation predictions in a consistent format that facilitates further analysis. To enable investigators to use a single format with a wide range of simulations, this format must be able to capture multidimensional data. To help investigators interpret simulation results, this format must also be able to capture metadata about the semantic meaning and provenance of simulation results, such as labels for each axis and labels for each slice of each axis.

SED-ML's report class can describe the data sets that simulation experiments should generate, including the specific predictions (e.g., trajectories of species produced by a continuous kinetic simulation or reaction fluxes produced by a flux balance simulation) that should be included in each data set. However, SED-ML does not detail the data structures that should be used to represent reports, which formats reports should be exported to, or what metadata should be bundled with such reports.

BioSimulators uses HDF5 to capture the values of reports specified in SED-ML documents. To ensure that simulation tools produce consistent results that can be consistently visualized and analyzed with downstream tools, we adopted the following conventions for encoding simulation results into HDF5 files. (a) Simulation tools should produce a single HDF5 file per COMBINE archive. These HDF5 files should contain the results of all of the reports specified in all of the SED-ML documents in the archive. (b) Each SED-ML report should be saved to a separate HDF5 data set. The key of each HDF5 data set for each SED-ML report should be the concatenation of the location of its parent SED-ML document within its parent COMBINE archive and the id of

the SED-ML report. (c) The values of SED-ML reports should be encoded into multidimensional HDF5 data sets. The first dimension (rows) of these HDF5 data sets should capture the SED-ML data sets of each SED-ML report. For simulation experiments that involve repeated SED-ML tasks, the next dimensions of these HDF5 data sets should capture successive layers of repeated tasks and their subtasks; HDF5 datasets should use two dimensions to capture each pair of layers of repeated tasks and their subtasks. The final dimensions of these HDF5 data sets should capture the independent dimensions of each atomic SED-ML simulation task (e.g., time for non-spatial kinetic simulations; time, x, y, and z coordinates for spatial kinetic simulations). (d) Before concatenating the values of the SED-ML data sets of a SED-ML report into a multidimensional matrix, values that have different shapes should be right-padded with 'NaN' into a common shape. The number of dimensions of this common shape should be equal to the maximum number of dimensions of the values of the individual SED-ML data sets. The length of each dimension of this common shape should be equal to the maximum length of the dimension over the values of the individual SED-ML data sets. (e) Simulation tools should encode metadata about the value of each SED-ML report into the attributes of the corresponding HDF5 data set. The ids, labels, shapes, data types of the slices of the first dimension of each HDF5 data set should be encoded into HDF5 attributes whose keys are 'dataSetIds,' 'dataSetLabels,' 'dataSetShapes,' and 'dataSetDataTypes' and whose values are arrays of the ids, labels, shapes, and NumPy dtypes of the SED-ML data sets of the corresponding SED-ML report.

### S2.3 Visualizations of simulation results: PDF and other domain-independent formats

Frequently, investigators use plots to gain insights from simulation predictions. SED-ML's plot classes can describe plots that simulation experiments should generate. However, SED-ML does not detail the format in which plots should be exported. To enable simulation tools to produce plots consistently, BioSimulators stipulates that simulation tools should export SED-ML plots in PDF format, that these PDF plot files should be bundled into a zip archive, and that the PDF files should be saved to paths within the zip archives equal to the concatenation of the location of their parent SED-ML documents within their parent COMBINE archives and their SED-ML ids.

By requiring simulation tools to produce consistent reports of simulation results, BioSimulators also makes it easier for investigators to use other tools to produce additional types of plots that cannot be described with SED-ML. In particular, we encourage investigators to visualize simulation predictions using tools such as ggplot2 (115), Vega (111), and Vega-Lite (116) that enable transparent, reusable data visualizations.

### S2.4 Logs of the execution of simulations: New YAML schema

As simulation experiments continue to grow more complex, it will be important for investigators to be able to quickly pinpoint problems with specific simulations within potentially large experiments that involve many individual simulations. To help investigators debug simulation experiments, we developed a new YAML schema for logs of the execution of COMBINE archives. This format can capture granular metadata about the execution of each simulation and output in each SED-ML document in a COMBINE archive. This includes the status of each task (scheduled, running, succeeded, failed, or skipped); the standard output and error of each completed task; and the wall

time of each completed task. For skipped and failed tasks, the schema can also capture the reason for their skip or failure.

In addition, the schema can capture the algorithm that the simulation tool executed for each SED-ML simulation, the function that the tool used to execute the simulation, and the arguments that the tool executed this function with. When SED-ML files specify classes of algorithms rather than particular algorithms (e.g., a Gillespie-like method rather than the Gibson-Bruck Next Reaction Method) or the simulation tool supports different algorithms than those specified in the SED-ML files (e.g., CVODE versus LSODA), these logs record the specific algorithms that the simulation tool executed for each simulation task. We believe this information makes the execution of simulations more transparent to investigators, which in turn, we anticipate will help investigators reproduce and debug simulations.

### S2.5 Simulation tools: Containerized command-line programs

BioSimulators standardizes simulation tools as Docker images whose entry points are standardized command-line programs that have two primary input arguments for (a) the path to the standardized simulation project in COMBINE archive format that the simulation tool should execute and (b) the path to save its output reports, plots, and logs in HDF5, PDF, and BioSimulators' YAML schemas.

To ensure that simulation tools can be run on high-performance computing (HPC) systems, we encourage developers to create Docker images that can be converted to the Singularity Image Format (SIF; 117). In particular, developers should avoid granting permissions to specific users, and developers should avoid installing software into $HOME and $TMP, paths that HPC systems often map to user home and temporary directories.

For broader compatibility beyond BioSimulators, we also encourage developers to use BioContainers (118) and Open Container Initiative (OCI) labels to encapsulate basic metadata about tools into their Docker images. These labels enable images to capture basic metadata, such as the name and version of the software in the image, a URL that contains further information about the software in the image, and contact information for the maintainer of the image.

### S2.6 Lower level simulation capabilities: Python APIs

To help investigators develop containerized command-line interfaces to simulation tools, we developed a convention for consistent Python APIs for simulation tools; BioSimulators-utils, a Python library for building such APIs; and a template for creating a containerized command-line interface from such an API. These conventions, library, and template are particularly useful to developers of simulation tools that are implemented in Python or provide Python interfaces. Due to the popularity of Python in biological modeling, this encompasses the majority of simulation tools, including 17 (80%) of the standardized tools presently registered with BioSimulators.

Importantly, these Python APIs also provide investigators consistent programmatic access to simulation capabilities, including both high-level access to the execution of entire COMBINE archives and lower-level access to the execution of individual simulation tasks. For example, these APIs could be used to develop web applications for interactively executing simple simulations and visualizing their results, or to combine multiple simulations of individual scales into multiscale simulations, such as using the Vivarium simulation composition framework (119, 120).

These Python APIs and the command-line programs built on top of them are available from PyPI at https://pypi.org/user/biosimulators.

### S2.7 Capabilities of simulation tools and the results of their verification: New JSON schema

Due to the numerous modeling formats, modeling frameworks, and simulation algorithms used in biomodeling, it is often difficult to find simulation tools that can execute specific simulations. To help investigators find simulation tools with specific capabilities (e.g., tools that support specific model formats, modeling frameworks, and/or simulation algorithms), we developed a JSON schema for capturing the capabilities of simulation tools. This format uses EDAM concepts to describe the model formats that tools support, SBO concepts to describe the modeling frameworks that tools support, KiSAO concepts to describe the simulation algorithms that tools support and the parameters that tools support for each algorithm, and SIO concepts to describe the dependent variables of each algorithm. The format can also capture the data type of each algorithm parameter, the default value of each parameter, and, optionally, a recommended range for the value of each parameter. To help investigators use simulation tools to execute SED-ML, the schema can also capture the SED-ML targets that simulation tools support for model changes and observables.

The schema can also capture a variety of metadata about a simulation tool. This includes the location of the Docker image for the tool, the version of the tool, a description of the tool, URLs and references that have additional information about the tool and each algorithm, the interfaces that the tool provides (e.g., API, console, desktop), the programming languages that the tool provides APIs for, the operating systems that the tool supports, the dependencies of the tool, the license for the tool, the authors of the tool and their Open Researcher and Contributor IDs (ORCIDs) (121), and the funding agencies which supported the development of the tool.

In addition, the schema can capture information about how the capabilities of a simulation tool were verified. Specifically, the schema can capture the results of the test cases of our test suite for simulation tools. For each test case, the schema can capture the id of the case; whether the simulation tool passed the case, failed the case, or the case was skipped because the tool is not expected to have the capabilities that the case probes; the standard output and error generated by the execution of the case; and the duration of the execution of the case. For failed cases, the schema can also capture the reason why the case failed. Similarly, the schema can capture the reasons why cases were skipped.

## S3 Foundational tools for validating simulation projects and simulation tools

To help investigators use the conventions described in the previous section, we also developed several tools for validating that simulation projects and simulation tools are consistent with these conventions.

## S3.1 Validation of simulation experiments described with SED-ML

To help investigators find errors in SED-ML documents and use SED-ML consistently, we developed a set of rules for validating SED-ML documents and implemented them into a Python API and command-line program with the BioSimulators-utils package described below. For example, these rules enable BioSimulators-utils to identify invalid relationships among SED-ML entities, invalid model changes such as using incompletely specified or invalid XPaths, and infinite repeated tasks. The rules also enable BioSimulators-utils to provide investigators warnings about potential mistakes, such as reports with repeated row headings and empty plots that lack curves.

## S3.2 Validation of metadata about simulation projects described with RDF-XML

To ensure investigators annotate simulation projects correctly, consistently, and thoroughly, we developed a program for validating metadata about simulation projects encoded into Resource Description Framework - Extensible Markup Language (RDF-XML) files. This program uses libOmexMeta (122) to validate that files are syntactically consistent with the RDF-XML format, uses the Identifiers.org registry of namespaces (123) to validate that each Identifiers.org identifier is syntactically valid within its namespace, and evaluates whether the file provides the minimum required metadata, such as a title for the parent COMBINE archive.

## S3.3 Validation of entire simulation projects

To help investigators develop COMBINE archives that are consistent with BioSimulators' conventions, we developed a program for validating entire COMBINE archives, including the manifests of the archives, the SED-ML files in the archives, the models involved in SED-ML files, metadata encoded in RDF-XML files in archives, and images referenced by those RDF-XML files. This program integrates validation performed by several individual tools into a single interface. This includes using libCOMBINE to validate the manifests in archives; using the programs described above to validate SED-ML files and metadata about simulation programs; and using libCellML, libSBML, pyBioNetGen, pyNeuroML (22), the Python interfaces to GINsim (124) and Smoldyn (125), and XPP (52) to validate the models involved in the SED-ML files in archives.

## S3.4 Validation of logs of the execution of simulation projects

To ensure that simulation tools produce consistent logs of the execution of simulation projects, we developed a program to validate such logs. This program checks that logs are syntactically consistent with the schema for logs and that each referenced KiSAO id is an id of an algorithm. This program is available through BioSimulators' REST API.

## S3.5 Validation of the specifications of the capabilities of simulation tools

To help investigators document the capabilities of their tools, we developed a program for validating the specifications of the capabilities of simulation tools. For example, the program checks that

specifications are syntactically consistent with the schema for the specifications of simulation tools, that each annotated algorithm has a unique KiSAO id, that each annotated parameter of each algorithm has a unique KiSAO id, and that the default value of each parameter is consistent with its annotated data type. This program is available through BioSimulators' REST API.

### S3.6 Validation of containerized interfaces to simulation tools

To ensure that simulation tools execute simulations consistently with BioSimulators' conventions (interpret SED-ML consistently and produce consistent outputs), we developed a test suite for validating simulation tools. The test suite is organized similarly to unit testing frameworks and test runners such as Python's unittest and pytest. Similar to such frameworks, the test suite is composed of several separate tests that verify the compatibility of individual aspects of simulation tools with BioSimulators' conventions. This includes tests that verify that (a) the entry points of containerized simulation tools are command-line programs; (b) these command-line programs support the arguments outlined above, correctly execute simulation experiments described with model formats such as SBML and KiSAO concepts in SED-ML documents in COMBINE archives, produce results in BioSimulators' HDF5 schema, produce plots in PDF format, and produce logs in BioSimulators' YAML schema; and (c) interfaces to simulation tools are annotated using BioSimulators' JSON schema.

The test cases that verify support for SED-ML, KiSAO, OMEX, and the COMBINE archive format execute example COMBINE archives and check that the expected reports and plots are created with the expected file names, shapes, values, and metadata. Each test case uses one or more example COMBINE archives that probe support for one or more specific features of SED-ML, HDF5, OMEX, or the COMBINE archive format. The most basic test case checks that the simulation tool correctly produces an HDF5 report of the results of the execution of a COMBINE archive that contains a single SED-ML file that describes a single simulation task and a single report. More advanced test cases check that simulation tools support additional features of SED-ML, HDF5, and COMBINE archives, such as multiple SED-ML files per COMBINE archive, models that must be resolved from URLs, model changes, multiple simulation tasks per SED-ML document, repeated tasks, algorithm parameters, and multiple reports and plots.

To minimize the number of manually curated COMBINE archives needed to test simulation tools, the test suite computationally generates COMBINE archives that are appropriate for testing each simulation tool from a small set of curated archives based on published simulation experiments. These curated archives contain individual SED-ML documents that describe individual simulation tasks and reports. First, the test suite uses the annotation of the simulation tool to determine which curated archives the tool should be able to execute. Second, the test suite uses this subset of archives as the basis for additional archives that test more advanced features of SED-ML, HDF5, and COMBINE and their combinations. For example, the test suite can use a curated COMBINE archive to create an additional archive that tests for support for multiple simulation tasks per SED-ML document by copying the simulation task in the curated archive, assigning it a distinct identifier, and appending data sets for the second simulation task to the report described in the curated archive.

Importantly, this design makes it easy to extend the test suite to additional model formats, modeling frameworks, and simulation algorithms. In most cases, only a single additional curated archive is needed to extend the test suite to an additional model format; at worst, extending the test suite

to an additional model format requires curating one COMBINE archive per unique combination of model format and simulation algorithm. For example, the test suite is able to test support for five flux balance simulation algorithms and three logical simulation algorithms with just one curated COMBINE archive each for the SBML-fbc and SBML-qual packages.

We provide two mechanisms for executing the test suite. First, developers can execute the test suite by submitting an issue to the BioSimulators GitHub repository that contains a URL where the specifications of their simulation tool can be retrieved. Developers can create issues either using the template available from the GitHub website or using the GitHub REST API. The latter enables developers to test their tools programmatically. The creation of each issue triggers a GitHub action which retrieves the specifications for the simulation tool, pulls the Docker image from the location described in the retrieved specifications, and executes the test suite on the Docker image. These actions communicate information about whether the simulation tool is valid via messages to the same GitHub issue. This cloud deployment enables developers to test their tools without installing any software. In addition, this cloud deployment enables the BioSimulators Team to monitor issues that developers are encountering and help them.

Alternatively, developers can install a command-line program for executing the test suite from PyPI and execute this program on their own machines. This program enables developers to either execute all of the tests or execute individual tests. The latter makes it easy for developers to quickly debug inconsistencies with BioSimulators' conventions by iteratively correcting issues with their simulation tool and using the test suite to verify their implementation.

# S4 Foundational tools for working BioSimulators' formats and ontologies

To help investigators work with the foundational formats and ontologies for BioSimulators, we developed two Python libraries: a library for querying the KiSAO ontology and identifying sets of similar algorithms and BioSimulators-utils, an integrated collection of methods for working with the foundational formats for BioSimulators. In addition, we have created several automated services for recommending simulation algorithms and simulation tools and we have created a web application which makes it easy for investigators to use the BioSimulators simulation tools to execute simulations.

## S4.1 KiSAO library: Python package for reasoning simulation methods

To help developers work with the KiSAO ontology, we developed a Python library for querying the ontology and identifying sets of similar algorithms. First, the library enables investigators to conduct basic queries, such as extracting the annotation of a specific algorithm, determining the parents of the algorithm, and identifying related algorithms that share common characteristics or parameters. Second, the library provides a method for identifying sets of similar algorithms and classifying their similarity into nine levels ranging from algorithms that execute mathematically equivalent algorithms (e.g., Gillespie's Direct Method (80) and the Gibson-Bruck Next Reaction Method (79)) to algorithms that differently approximate the same mathematics (e.g., CVODE (69) and Euler's method) to algorithms that use different mathematics to predict the same observable (e.g., FBA and pFBA). These methods use the parent-child relationships (e.g., the Gibson-Bruck Next Reaction

Method is a child of the Gillespie-like class of simulation algorithms) and relationships among algorithms and characteristics of algorithms (e.g., the Gibson-Bruck Next Reaction Method performs mathematically-equivalent simulation to Gillespie's direct method; tau-leaping approximates Gillespie's direct method) encoded into the KiSAO ontology. On top of this method, the library provides another method for identifying the most similar alternative algorithm to a given algorithm among a set of algorithms. When users request simulations involving unsupported algorithms, the BioSimulators interfaces to simulation tools use this method to automatically identify the best alternative algorithm, notify the user that an alternative algorithm will be executed, execute the alternative algorithm, and log which algorithm was executed so that the user can transparently inspect what the simulation tool did. This method also provides users an option to control the degree to which algorithms can be substituted with alternative ones before errors are raised. This library is available at https://pypi.org/project/kisao.

## S4.2 BioSimulators-utils: Python package for building standardized interfaces to simulators

To help developers create standardized biosimulation software tools, we developed BioSimulators-utils, a high-level Python library for working with COMBINE archive, HDF5, OMEX, and SED-ML files and BioSimulators' schema for logs of the execution of COMBINE archives. BioSimulators-utils provides methods for reading and writing these formats, validating that documents are syntactically and semantically consistent with these formats, orchestrating the execution of SED-ML documents in COMBINE archives, and logging the execution of COMBINE archives. This includes methods for resolving models defined in SED-ML from local file paths, URI fragments, and external URLs; modifying models according to all of the types of changes that can be described with SED-ML; orchestrating the execution of individual simulation tasks with specific algorithms and specific values of their parameters; orchestrating the execution of repeated simulation tasks over all of the types of ranges that can be described with SED-ML; generating SED-ML reports and plots; saving reports to HDF5 files and saving plots to zip archives of PDF files; and compiling logs of the execution of individual SED-ML tasks, reports, and plots into a single log for each COMBINE archive. BioSimulators-utils also provides methods for implementing command-line interfaces to simulation tools, pushing and pulling Docker images that contain standardized simulation tools to and from the BioSimulators Docker registry, and using these Docker images to execute COMBINE archives. Further information about BioSimulators-utils is available at https://biosimulators.github.io/Biosimulators_utils/.

Several lower-level libraries are also available for working with the individual formats and ontologies used by BioSimulators. This includes libCOMBINE for reading and writing COMBINE archives and OMEX files; libSEDML and jlibSEDML for reading and writing SED-ML documents and executing basic operations described by SED-ML documents; libKiSAO for querying HDF5 (126); and libHDF5, H5Py, hdf5r, rhdf5, and JHDF5 for reading and writing HDF5 files.

## S4.3 Automated services for recommending simulation algorithms and tools

To help investigators identify appropriate tools for their work, we have also developed four modes of automatically recommending simulation algorithms and tools. These services utilize the KiSAO ontology of simulation algorithms, the methods in the KiSAO Python library described above, and the BioSimulators database of the capabilities of simulation tools.

- As described above, our KiSAO library and REST API provide methods for identifying all of the algorithms that are similar to a given algorithm and the degree of similarity of each alternative algorithm.
- BioSimulators provides a web form for using this information to get recommendations for tools for executing particular algorithms. First, the user selects a particular simulation algorithm that they would like to execute. Second, the form uses KiSAO to identify all of the similar algorithms and the degree of their similarity. Third, the form uses the BioSimulators database of the capabilities of simulation tools to determine the maximum degree of algorithmic similarity to which each simulation tool can execute the selected algorithm. Fourth, the form sorts the simulation tools by their maximal degree of algorithmic similarity. Finally, the form displays the most similar simulation tools and indicates the most similar algorithm that each tool supports.
- runBioSimulations provides a web form which can recommend appropriate simulation tools for a specific COMBINE archive. First, the user selects a COMBINE archive. Second, the form analyzes the archive to determine the simulation algorithms and model formats involved. Third, the user chooses the degree to which the specified simulation algorithms could be substituted to alternative methods. Fourth, the form uses the KiSAO ontology and the BioSimulators database of simulation tools to determine the tools which support the required model formats and which support simulation algorithms that are at least as similar to the specified simulation algorithms as the chosen minimal degree of similarity. Finally, the form reports the list of appropriate simulation tools to the user.
- As described above, each BioSimulators interface to a simulation tool automatically executes the most similar algorithms that it supports to the algorithms specified in the requested COMBINE archive. When simulation tools execute alternative algorithms, they warn users about this and log the algorithms that they executed. Users can also control the degree of dissimilarity at which an error rather than a warning should be raised.

In addition, the BioSimulators web application provides tools for comprehensively exploring the capabilities of all of the registered tools and identifying tools that support particular combinations of model changes, observables, simulation algorithms, algorithm parameters, model formats, and other attributes of simulation tools. For example, this can be used to identify all of the tools that support algorithms for a particular modeling framework.

### S4.4 runBioSimulations web application for executing simulations and visualizing and sharing their results

To make it easy for investigators to use BioSimulators simulation tools to execute simulations, we have also developed runBioSimulations (127), a web application that provides users a simple web interface where they can create, validate, and execute COMBINE/OMEX archives and visualize the results of their simulations. runBioSimulations also provides investigators capabilities to track their simulation runs and privately share simulation runs, such as with colleagues and peer reviewers.

## S5 Workflow for creating and registering standardized simulation tools

BioSimulators supports a straightforward workflow for creating standardized simulation tools and an automatable workflow for submitting and updating them to BioSimulators' registry (Figure S2). (1) Developers must map the inputs and outputs of their simulation tool to community conventions, including SED-ML and the COMBINE archive format. (2) Developers must encapsulate their tool into a command-line program in a Docker image and push the image to a public Docker image registry, such as Docker Hub or GitHub Container Registry. (3) Developers must document the capabilities of their tool, including the model formats, modeling frameworks, and simulation algorithms that it supports, in BioSimulators' JSON schema. (4) Developers must submit their simulation tool to BioSimulators. (5) BioSimulators will then verify the tool using a combination of automated testing and manual review. (6) BioSimulators will save valid tools to its database.

To maximize the community's ability to use BioSimulators to share information about simulation tools, we also welcome submissions of non-standard simulation tools. In this case, developers only need to submit metadata about their tool. Note, the BioSimulators curators cannot verify the capabilities of such non-standard simulation tools, and the BioSimulators website will inform the community that the capabilities of these non-standard tools have not been verified. This strategy makes it easy both for developers to contribute tools to BioSimulators and for users to find and use verified tools.

This section summarizes the process for standardizing a simulation tool and submitting it to BioSimulators. More detailed guides to standardizing simulation tools and submitting them to BioSimulators are available at https://docs.biosimulations.org. The BioSimulators Team is also available to help investigators develop standardized interfaces to their tools. We encourage investigators to contact the BioSimulators Team at info@biosimulators.org support.

### S5.1 Map the inputs and outputs of the simulation tool to community conventions

To provide the community with standardized access to a simulation tool, first, the developers must map its inputs and outputs to several community formats. The input to each simulation tool must be a COMBINE archive (105). Simulation tools must be able to execute simulation experiments described in SED-ML in COMBINE archives involving models described in formats such as BNGL, CellML, or SBML and algorithms indicated using KiSAO concepts. Simulation tools must save all of the results of all of the reports in the SED-ML documents in a COMBINE archive to a single HDF5 file. Simulation tools should save all of the plots described in the SED-ML documents in COMBINE archives in PDF format. Furthermore, simulation tools should bundle these PDF files into a single zip archive file. Within each HDF5 file and zip archive, each report and plot should be saved to a path equal to a tuple of the location of its parent SED-ML document within its parent COMBINE archive and the id of the report or plot. In addition, simulation tools are encouraged to output logs of the executions of COMBINE archives in BioSimulators' YAML schema.

The BioSimulators-utils Python package provides developers with several high-level methods for working with COMBINE archives, SED-ML documents, HDF5 files, and zip archives. A template for using BioSimulators-utils to build a standardized interface to a simulation tool is available from

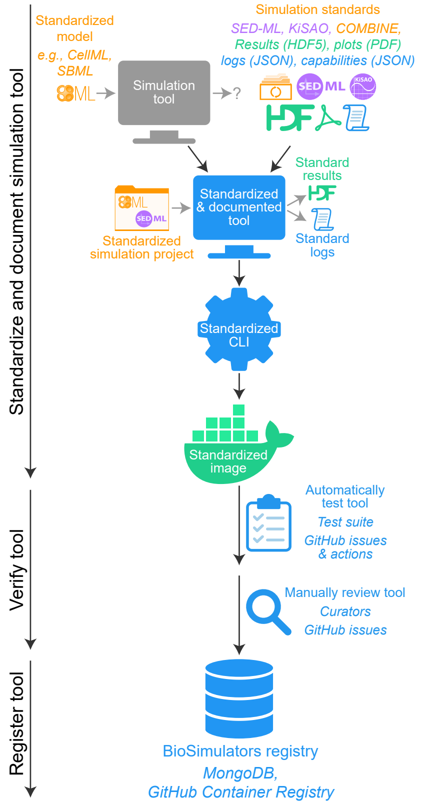


**Figure S2: BioSimulators supports a straightforward workflow for creating and registering standardized simulation tools for a broad range of model formats, modeling frameworks, and simulation algorithms.** (1) Developers map the inputs and outputs of their tools to formats such as SED-ML, BioSimulators' HDF5 schema, and BioSimulators' YAML schema for logs of the execution of simulations. (2) Developers create standardized command-line interfaces for tools and containerize them. (3) Developers curate the capabilities of their tools. (4) Developers submit their tools to BioSimulators. Developers can submit tools manually or programmatically. The latter makes it easy for developers to automatically push new versions of their tools to BioSimulators. (5) BioSimulators' test suite automatically verifies that tools execute simulations consistently with BioSimulators' conventions. (6) BioSimulators' curators check that tools are annotated accurately and thoroughly. (7) The BioSimulators test suite and team communicate issues with tools to their developers. (8) Once tools are valid, their Docker images and metadata about their capabilities are automatically saved to BioSimulators' database. Importantly, this workflow supports a broad range of model formats, modeling frameworks, and simulation algorithms by abstracting their details into COMBINE archives, ontology concepts, and Docker images. Grey indicates simulation tools. Orange and purple indicate existing modeling formats and ontologies that BioSimulators uses for the inputs to tools; orange indicates resources that BioSimulators uses without modification; purple indicates resources that we refined or expanded to enable BioSimulators. Green indicates existing domain-independent formats that we customized for simulation tools and their outputs. Blue indicates BioSimulators' new schemas and other new resources that standardize tools and logs of their execution.

the BioSimulators GitHub organization.

## S5.2 Encapsulate the tool into a command-line program

To make it easy for investigators to execute their simulation tool, developers must next encapsulate their tool into a command-line program. Command-line programs for simulation tools should have two arguments: a path to the COMBINE archive that the tool should execute and the path where the simulation tool should save the outputs of the execution of the archive (a single HDF5 file

with the results of each report described in each SED-ML document in the COMBINE archive, a zip archive with a PDF file for each plot described in each SED-ML document in the COMBINE archive, and a YAML-formatted log of the execution of the archive).

### S5.3 Encapsulate the command-line interface to the tool into a Docker image

To make it easy to execute simulation tools across a wide range of computational environments, the developers must next encapsulate the command-line program for their tool and its dependencies into a Docker image. To facilitate usage in HPC clusters, developers should create Docker images that are compatible with Singularity. The entry point for the image must be the command-line program for the tool. BioSimulators also encourages developers to use BioContainers (118) and Open Container Initiative (OCI) labels to annotate their images (e.g., the name, version, license, and developers of the software in the image).

### S5.4 Push the Docker image for the tool to a public Docker registry

To make the Docker image for their simulation tool accessible to BioSimulators, developers should next push their image to a public Docker registry such as Docker Hub, GitHub Container Registry, or Quay. Developers should use distinct tags to manage multiple Docker images for multiple versions of their tool.

### S5.5 Annotate the capabilities of the simulation tool and other metadata

Next, developers must use BioSimulators' JSON schema and several community ontologies to describe the capabilities of their simulation tool, including the model formats (e.g., BNGL, SBML), modeling frameworks (e.g., flux balance, logical, discrete kinetic), and simulation algorithms (e.g., flux balance analysis, Gillespie's direct method) that the simulation tool supports; the independent and dependent simulation variables that each simulation algorithm supports; the parameters of each simulation algorithm; and the data type, default value, and recommended range of values of each parameter. Developers must use concepts in the EDAM ontology to describe the model formats that their tool supports. Developers must use the SBO ontology to describe the modeling frameworks that their tool supports. Developers must use the KiSAO ontology to describe the simulation algorithms that their tool supports and their parameters. Developers must use the SIO ontology to describe the independent variables (e.g., time, x-coordinate) that each algorithm supports. BioSimulators allows developers to describe the dependent model variables that can be recorded for each simulation algorithm and the targets that users can use to record specific variables in conjunction with model variables in SED-ML documents. For XML-based model formats (e.g., CellML, SBML), BioSimulators recommends that the targets of model variables be their XML XPATHs in the XML descriptions of their parent models.

Developers can also use BioSimulators' schema for the capabilities of simulation tools to capture metadata about their tools, such as the interfaces (e.g., command-line, desktop) that tools provide, the operating systems that tools support, the programming languages that tools provide libraries for, hyperlinks to further information about tools, licenses for tools, citations for tools, and the authors of tools. Developers can use SPDX identifiers to describe the licenses of their tools.

## S5.6 Verify that the simulation tool is packaged and annotated consistently with BioSimulators' conventions.

Next, developers can use the BioSimulators test suite described above to verify that their simulation tool and its specifications are consistent with BioSimulators' conventions. As described above, developers can execute the test suite on their own machines using its command-line interface or use the deployment of the test suite that is hosted through GitHub issues and actions as described below.

## S5.7 Submit the tool to the BioSimulators registry

Next, developers can submit their tool to BioSimulators by creating an issue for the BioSimulators GitHub repository, whose body contains a URL where the specifications for the simulation tool can be retrieved. Developers can create issues either using the issue template available through the GitHub website or using the GitHub REST API. Similar to the simulator verification system described in the previous section, the creation of each issue triggers a GitHub action which retrieves the specifications of the simulation tool, pulls the Docker image for the simulation tool from the location indicated in the specifications, uses the test suite to verify the Docker image for the simulation tool, and saves a report of the verification of the tool as a JSON-formatted file. If the simulator fails the test suite, the test suite reports its failures via messages to the same GitHub issue. After addressing the issues identified by the test suite, developers can re-start this submission workflow by editing the body of the GitHub issue. This will trigger the test suite to review the simulator again.

The first time each simulation tool passes the test suite, the test suite will assign the BioSimulators Team to review the simulator manually. The BioSimulators Team will then use the same GitHub issue to discuss any issues that the developers must address to make their simulation tool consistent with BioSimulators' conventions. Once the developers have addressed the issues identified by the BioSimulators Team, the BioSimulators Team will add the 'Approved' label to the GitHub issue. This will trigger another GitHub action which will add a standardized tag to the Docker image for the simulator, push this tag to BioSimulators' Docker image registry so that BioSimulators retains a copy of the image for the community, and use BioSimulators' API to save the capabilities of the simulator and its verification record to BioSimulators' database. Subsequent versions of the same simulation tool will be automatically approved and immediately saved to BioSimulators' database once they pass the test suite.

We believe this quality control scheme has several advantages. First, this combination of automated tests executed using GitHub actions and manual review organized with GitHub issues both ensures that the majority of our review is fair and objective, and it enables us to manually review complex issues that are challenging to verify computationally, such as the completeness of the annotation of the capabilities of simulation tools. This combination also makes it possible for us to rigorously review each version of each simulation tool with a modest amount of effort. Second, the GitHub API makes it easy for developers to programmatically submit each version of their tools to BioSimulators. Third, GitHub automatically tracks the provenance of each submission, including the people who submitted and reviewed each tool and when the simulator was submitted, reviewed, and approved. Fourth, the open nature of GitHub issues and actions makes the review of simulation tools transparent to the community. Fifth, we anticipate this system will make it easy for us to identify

issues that developers are struggling with and help developers resolve these issues.

### S5.8 Automate the submission of subsequent versions of the tool to BioSimulators

To enable the community to use specific versions of simulation tools, we encourage developers to submit each version of their simulation tools to BioSimulators. We recommend that developers achieve this by creating continuous deployment workflows that use the GitHub REST API to automatically create issues that submit their tool to BioSimulators each time they release a new version of their tool. An example GitHub actions workflow is available from the BioSimulators GitHub organization.

## S6 Architecture, implementation, testing, and deployment of BioSimulators

### S6.1 Architecture

BioSimulators is compromised of (a) a set of formats and specifications for the inputs and outputs of simulation tools which enable simulation tools to provide investigators a simple, consistent interface for executing simulations, (b) a library for working with these formats, including validating that documents are consistent with these formats, (c) a schema for metadata about the capabilities of simulation tools, (d) a test suite for verifying that simulation tools follow these conventions, (e) a web service for running this test suite in the cloud, (f) a database of metadata about simulation tools, (g) a registry for Docker images for standardized simulation tools, (h) an OpenAPI-compliant REST API for modifying and querying the database, (i) a web application for manually reviewing simulation tools submitted to the database, and (j) a graphical user interface (GUI) for browsing the database, getting recommendations for simulation algorithms and tools, and validating modeling projects (Figure S3).

### S6.2 Implementation, testing, and deployment

BioSimulators is implemented as a collection of modular components, each implemented with different tools as appropriate. We used GitHub to coordinate the development of these components.

#### S6.2.1 Schemas for the capabilities of simulation tools, the verification of simulation tools, and logs of the execution simulation projects

We used NestJS and OpenAPI to define and document these schemas and implement methods for validating that documents are consistent with the schemas.

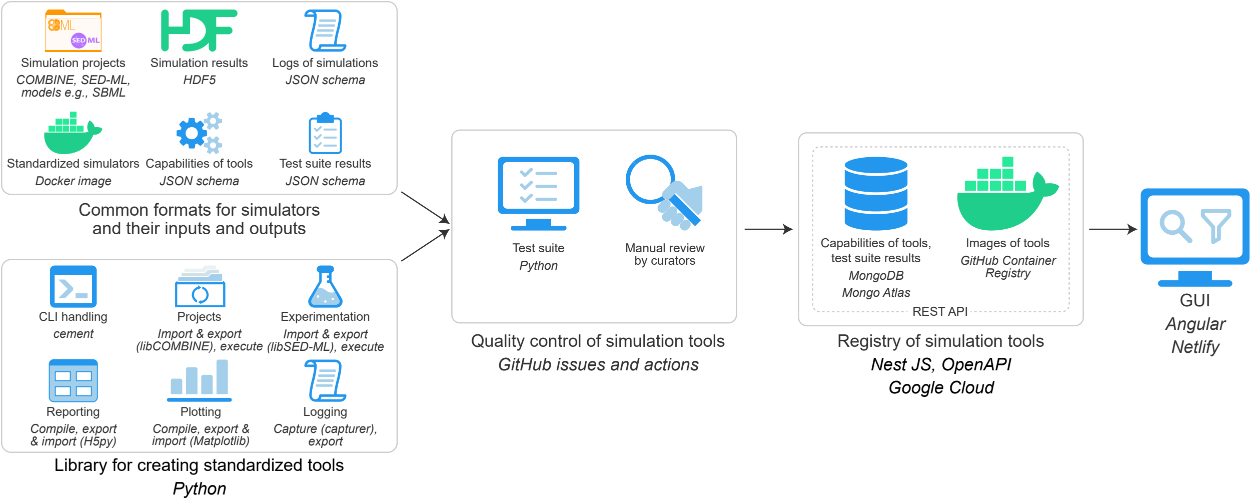


**Figure S3: BioSimulators' modular, standards-driven architecture enables BioSimulators to provide the community standardized tools for a wide range of model formats, modeling frameworks, and simulation algorithms.** The BioSimulators ecosystem includes a set of formats for simulation tools and their inputs and outputs; a library for creating standardized simulation tools that support these formats; a test suite for verifying that simulation tools execute these formats consistently; an open registry of standardized simulation tools and metadata about their capabilities; an online system for quality controlling submissions to the registry which employs this test suite and further manual review; and a GUI for browsing the registry, getting recommendations for simulation algorithms and tools, and validating modeling projects. Importantly, these components facilitate the standardization of simulation tools for a wide range of model formats, modeling frameworks, and simulation algorithms by abstracting their details into COMBINE archives, ontology concepts, and Docker images. Orange and purple indicate existing modeling formats and ontologies that BioSimulators embraces for the inputs to simulation tools; orange indicates resources that BioSimulators embraces without modification; purple indicates resources that we refined or expanded to enable BioSimulators. Green indicates existing domain-independent formats and resources that we customized for simulation tools and their outputs. Blue indicates new schemas and other resources that we developed to enable BioSimulators.

### S6.2.2 Schema for the results of SED-ML reports

The schema for the results of SED-ML reports is implemented as a set of guidelines for using HDF5. These guidelines simply outline the paths where the results of SED-ML reports should be located within HDF5 files and how metadata about SED-ML reports should be encoded into the attributes of HDF5 data sets. These guidelines are described in the BioSimulators documentation, and the test suite checks that simulation tools follow these guidelines.

### S6.2.3 KiSAO library

The KiSAO library is implemented in Python using the pronto library. The library was tested using pytest, and the coverage of the tests was evaluated with CodeCov.

### S6.2.4 BioSimulators-utils library

BioSimulators-utils is implemented in Python using several packages including capturer, H5py, libCellML, libCOMBINE, libOmexMeta, libSBML, libSEDML, lxml, Matplotlib (128), NetworkX (129), NumPy (130), pyBioNetGen, pyNeuroML, and XPP. BioSimulators-utils was tested using

pytest, and the coverage of the tests was evaluated with CodeCov.

#### S6.2.5 Test suite for interfaces to simulation tools

The test suite is implemented in Python using BioSimulators-utils and several additional packages, including capturer and Cement. The test suite was tested using pytest, and the coverage of the test suite was evaluated with CodeCov. As described above, the cloud version of the test suite is deployed as a GitHub action that is triggered by the creation, editing, and tagging of issues for the BioSimulators GitHub repository.

#### S6.2.6 Database of the capabilities of simulation tools and their verification

We implemented the database using MongoDB and used Mongoose and NestJS to define the schema for the database and validate submissions to the database. The database is deployed using MongoDB Atlas.

#### S6.2.7 Docker registry of containerized simulation tools

BioSimulators uses the GitHub Container Registry to store a copy of each Docker image accepted to the BioSimulators registry. This design ensures that these Docker images will remain accessible to the scientific community even if the original authors delete their copies of the images.

#### S6.2.8 REST API

We implemented the API in TypeScript using NestJS, Auth0, and OpenAPI. We used NestJS to organize the API, we used Auth0 to manage access to the API, and we used OpenAPI to document the API. The API is deployed using a Kubernetes cluster running on Google Cloud.

#### S6.2.9 Graphical user interface (GUI)

We implemented the GUI in TypeScript using the Angular framework and the Material user interface component library. We tested the GUI using Jest. The GUI is deployed using Netlify. Errors encountered during deployment are logged using Google Cloud Error Reporting.

## S7 Case study: Assessing the practical reusability of published simulation experiments to individual investigators

In this section, we present a case study that illustrates the utility of the capability to use BioSimulators to execute a broad range of simulations.

A promising way to create comprehensive, predictive simulations is to combine simulations of individual subsystems developed by individual research groups. As a first step, this requires investigators

to be able to reuse individual simulations.

Recently, the BioModels curators, who have experience reusing models and the time to debug problems, found that they could reuse 61% of models reported in publications (131). However, individual investigators need to be able to reuse models quickly with modest effort. Furthermore, investigators need to be able to use simulations in addition to models. Due to greater support for community model formats among simulation software tools than for SED-ML, the findings of studies such as Tiwari et al. may not reflect the ability of individual investigators to reuse published simulation experiments.

To estimate the reusability of published simulation experiments to individual investigators, we used the standardized simulation tools available through BioSimulators to execute the curated simulation experiments available from BioModels (132), one of the largest collections of published simulation experiments. BioSimulators is ideal for this purpose because BioSimulators makes it easy to execute a broad range of simulation experiments. First, we used BioModels' REST API to determine which BioModels entries include simulation experiments in SED-ML format. As of the time of this study (Spring 2020), we determined that 214 BioModels entries included simulation experiments. Second, we used BioModels' REST API to download these experiments as COMBINE archives that contain SED-ML files. Third, we used the standardized simulation tools registered with BioSimulators to execute these experiments. This revealed that only two ($< 1\%$) of these simulation experiments could be executed.

Fourth, we used the execution logs of these simulation experiments to dissect the proximate reasons why most of these COMBINE archives cannot be executed. We found that 84% of the SED-ML files in the COMBINE archives have broken references to model files (the model in the COMBINE archive is located at a different path than that indicated in the SED-ML file) and that 15% of the SED-ML files in these archives are syntactically invalid, most frequently due to lacking required attributes, having duplicate values of attributes which must be unique, and having attributes with invalid values. We also found additional issues that prevent the execution of an additional 1% of these simulation experiments.

We also examined the results of the two COMBINE archives that did successfully execute to gain insights into best practices for publishing reproducible simulation experiments. This revealed another issue, that the simulation experiments in one of the COMBINE archives reference models outside the archive. While this mistake enables simulation tools to execute this COMBINE archive, this design is inconsistent with BioModels' goal of exporting COMBINE archives that standalone, containing all of the information required to execute the simulation experiment.

Fifth, we used BioSimulations-utils to dissect these COMBINE archives and identify additional issues beyond those identified above that would prevent their successful execution. This revealed additional issues with 20% of the simulation experiments. Many of these issues fall into the same categories outlined above. In addition, some of the simulation experiments have invalid entity references (e.g., references to undefined models).

Going forward, we aim to help the developers of BioModels address these issues.

## S8 Comparison of BioSimulators with other resources

Several resources have been developed to help developers build and share interoperable simulation tools and help investigators find and use these tools. This includes formats such as SBML and SED-ML which enable investigators to execute the same models and simulations with multiple simulation tools; formats such as HDF5, netCDF (133), N5, NuML (134, 135), and Zarr which can capture simulation results; test suites that help developers build simulation tools that generate consistent predictions (136, 137); registries of scientific software tools that help investigators find specific tools for specific tasks such as bio.tools (138), CoLoMoTo Docker (124), the PETab (139) feature support guide, the SBML Software Guide, and SciCrunch (140); repositories of scientific software packages such as Bioconda (141), BioContainers (118), and Dockstore (142); management systems for plugins to platforms such as BioUML (3), Galaxy (143), and KBase (144); and web-based simulation tools such as The Cell Collective (49), JWS Online (24), SBMLWebApp (145), and StochSS (19).

Nevertheless, it has remained difficult for investigators to share, reuse, and compose models and simulations, especially across biological subsystems and scales, due to gaps within and among these resources. For example, prior to BioSimulators EDAM lacked concepts for several popular modeling formats, KiSAO lacked concepts for many popular simulation algorithms; SED-ML did not specify a specific format for the results of simulation experiments; only a few simulation tools could execute simulation experiments described with SED-ML; simulation tools provided heterogeneous interfaces for executing simulation experiments; the scientific software registries were siloed from ontologies such as KiSAO that can capture the capabilities of simulation tools with sufficient granularity to help investigators find tools that can execute specific algorithms; the software registries that have metadata about the capabilities of simulation tools were siloed from registries of packages and Docker images of these tools; the test suites that help developers identify inconsistencies between their simulation tools and community model formats were siloed from the software registries that help investigators find tools; and the test suites did not verify that simulation tools execute simulation experiments described in SED-ML consistently.

BioSimulators unifies this siloed landscape by filling in gaps in these resources and linking them together. (a) We contributed numerous additional concepts to the EDAM ontology of scientific formats, the KiSAO ontology of simulation algorithms and their parameters, and the SIO ontology of physical, processual, and informational entities. (b) We filled in gaps in the specifications of SED-ML with a variety of concrete guidelines, such as for using XML namespaces and XPATHs for describing targets to model elements. (c) We introduced a schema for the results of simulation experiments, a schema for logs of the execution of simulation experiments, specifications for command-line interfaces and Docker images for simulation tools, a schema for the capabilities of simulation tools, and a test suite for verifying that simulation tools execute SED-ML documents and COMBINE archives consistently. (d) We created a new registry for simulation software tools. (e) We linked our registry to EDAM and KiSAO to capture granular information about the capabilities of each tool, we integrated our registry with a Docker image repository to capture Docker images of each tool, and we combined our registry with automated testing and manual review to quality control submissions to our registry. Furthermore, we deployed this testing in the cloud to enable developers to routinely push new versions of their tools to BioSimulators.

## S9 Availability of BioSimulators

The various components of BioSimulators are freely and openly available at the locations listed below. All of these components are available under the MIT license, except some of the standardized simulation tools, which are available under the open-source licenses noted at https://biosimulators.org.

- GUI for browsing the registry and getting recommendations for tools: https://biosimulators.org
  - Source code: https://github.com/biosimulations/Biosimulations
  - Tutorial and help: https://docs.biosimulations.org
  - Guide to contributing: https://github.com/biosimulations/Biosimulations

- REST API: https://api.biosimulators.org
  - Source code: https://github.com/biosimulations/Biosimulations
  - Documentation: https://api.biosimulators.org
  - Guide to contributing: https://github.com/biosimulations/Biosimulations

- Backend database
  - Source code: https://github.com/biosimulations/Biosimulations
  - Guide to contributing: https://github.com/biosimulations/Biosimulations

- Tools for validating simulation projects and their components
  - Web application: https://biosimulators.org
  - REST API: https://combine.api.biosimulations.org
  - Command-line application: https://pypi.org/project/biosimulators-utils
  - Python API: https://pypi.org/project/biosimulators-utils
  - Source code: https://github.com/biosimulators/Biosimulators_utils
  - Documentation: https://biosimulators.github.io/Biosimulators_utils
  - Guide to contributing: https://github.com/biosimulators/Biosimulators_utils

- Interfaces to simulation tools
  - Docker images: https://github.com/biosimulators
  - Command-line programs: https://pypi.org/user/biosimulators
  - Python APIs: https://pypi.org/user/biosimulators
  - Source code: https://github.com/biosimulators
  - Documentation and guides to contributing: https://docs.biosimulations.org

- Library for creating standardized interfaces to simulation tools (BioSimulators-utils)
  - Python package: https://pypi.org/project/biosimulators-utils
  - Source code: https://github.com/biosimulators/Biosimulators_utils
  - Documentation: https://biosimulators.github.io/Biosimulators_utils
  - Guide to contributing: https://github.com/biosimulators/Biosimulators_utils

- Template for a standardized interface to a simulation tool
  - Source code: https://github.com/biosimulators/Biosimulators_simulator_template
  - Documentation and guide to contributing: https://github.com/biosimulators/Biosimulators_simulator_template

- Test suite for verifying that simulation tools are consistent with BioSimulators' conventions

  - Cloud deployment: https://github.com/biosimulators/Biosimulators/issues/new/choose
  - Python package: https://pypi.org/project/biosimulators-test-suite
  - Source code: https://github.com/biosimulators/Biosimulators_test_suite
  - Documentation: https://biosimulators.github.io/Biosimulators_test_suite
  - Guide to contributing: https://github.com/biosimulators/Biosimulators_test_suite

- Library for working with the KiSAO ontology of simulation algorithms
  - Python package: https://pypi.org/project/kisao
  - Source code: https://github.com/SED-ML/KiSAO
  - Documentation: https://github.com/SED-ML/KiSAO
  - Guide to contributing: https://github.com/SED-ML/KiSAO

## S10 Community feedback, input, and contributions to BioSimulators

We welcome community feedback, input, and direct contributions to all of the components of BioSimulators. Simulation tools can be contributed by creating issues for the BioSimulators GitHub repository, https://github.com/biosimulators/Biosimulators. Guides to contribute to the other components of BioSimulators are available at the links above. We also encourage developers to use the BioSimulators API to develop additional applications.

## S11 Author contributions

**Agnew:** Conventions for Python APIs for simulation tools and simulation results.

**Andrews:** Interface to Smoldyn and its capabilities.

**Anwar:** Interface to COBRApy.

**Beber:** Interface to COBRApy.

**Bergmann:** Enhancement of SED-ML and libSED-ML, bug fixes to libCOMBINE, bug fixes to COPASI, development of the interface to COPASI, specifications of COPASI.

**Blinov:** Conception, interface to Virtual Cell and its specifications.

**Brooks:** Inteface to OpenCOR.

**Brusch:** Specifications of Morpheus.

**Calzone:** Specifications of MaBoSS.

**Choi:** Interface to tellurium.

**Cooper:** Bug fixes to GillesPy2, interface to GillesPy2, automated releasing of GillesPy2.

**Detloff:** User interface.

**Drawert:** Bug fixes to GillesPy2, interface to GillesPy2, automated releasing of GillesPy2, capabilities of GillesPy2.

**Dumontier:** Ontology terms for describing the outputs of simulations in SIO.

**Ermentrout:** Interface to XPP and its specifications.

**Faeder:** Interface to BioNetGen, capabilities of BioNetGen, and example models.

**Freiburger:** Example models and data visualizations, conventions for simulation results and data visualizations.

**Fröhlich:** Interface to AMICI and its capabilities.

**Funahashi:** Interface to libSBMLsim and its capabilities.

**Garny:** Interface to OpenCOR and its capabilities, validation of CellML files.

**Gennari:** Conventions for meta data about simulation projects and their components.

**Gleeson:** Interfaces to Brian 2, pyNeuroML, NEURON, and NetPyNe; validation for NeuroML and LEMS files.

**Goelzer:** Interface to RBApy and its capabilities.

**Goldberg:** Conception.

**Haiman:** Interface to MASSpy and its specifications.

**Hasenauer:** Interface to AMICI and its capabilities.

**Hellerstein:** Conventions for specifications of simulation tools.

**Hermjakob:** Curation of namespaces for annotating simulation projects.

**Hoops:** Capabilities of COPASI.

**Ison:** Curation of modeling and simulation formats in EDAM.

**Jahn:** Specifications of Morpheus.

**Jakubowski:** Conventions for Python APIs for simulation tools and simulation results.

**Jordan:** Curation of modeling and simulation formats.

**Kalaš:** Curation of modeling and simulation formats in EDAM.

**Karr:** Conception, conventions, ontologies, validation tools, interfaces to simulation tools and their specifications, backend, user interface, manuscript.

**König:** Improvements and clarifications to SED-ML, interface to tellurium.

**Liebermeister:** Interface to RBApy and its capabilities.

**Malik Sheriff:** Input to validation of simulation projects and user interface.

**Mandal:** Automated releasing of COBRPApy.

**Marupilla:** Interface to Virtual Cell, input into validation tools.

**McDougal:** Interface to XPP.

**Medley:** Interface to tellurium and its capabilities, conventions for specifications of simulation tools.

**Mendes:** Capabilities of COPASI.

**Moraru:** Conception, interface to Virtual Cell and its specifications, conventions, deployment.

**Müller:** Specifications of Morpheus.

**Myers:** Specifications of iBioSim.

**Naldi:** Interface to GINsim.

**Nguyen:** Validation of simulation projects.

**Nickerson:** Enhancement of SED-ML, interface to OpenCOR, validation of CellML files.

**Olivier:** Interfaces to CBMPy and PySCeS.

**Patoliya:** Curation of modeling and simulation formats in EDAM.

**Paulevé:** Interface to mPBN and its specifications, curation of algorithms and algorithm parameters for logical simulation.

**Petzold:** Interface to GillesPy2.

**Priya:** Curation of modeling and simulation formats in EDAM.

**Rampadarath:** Input into conventions for specifications of simulation tools and meta data about simulation projects.

**Rohwer:** Interface to PySCeS.

**Saglam:** Interface to BioNetGen, validation of BNGL files, example BNGL files.

**Sauro:** Conception, interface to tellurium and its specifications.

**Shaikh:** Conventions, backend, user interface, deployment, manuscript.

**Singh:** Interface to Smoldyn, specifications of Smoldyn, automated releasing of Smoldyn.

**Sinha:** Validation of NeuroML; interfaces Brian 2, pyNeuroML, NEURON, NetPyNe.

**Smith:** Conventions, enhancement of SED-ML, validation of SBML files and simulation projects, interface to tellurium and its specifications.

**Snoep:** Conventions for Python APIs for simulation tools.

**Sorby:** Validation of CellML files.

**Spangler:** Conventions for Python APIs for simulation tools.

**Starruß:** Specifications of Morpheus.

**Thomas:** Specifications of iBioSim.

**van Niekerk:** Conventions for Python APIs for simulation tools.

**Vasilescu:** Interface to Virtual Cell, input into validation tools.

**Weindl:** Interface to AMICI and its specifications.

**Wilson:** Deployment.

**Zhang:** Specifications of Simmune.

**Zhukova:** Curation of algorithms and algorithm parameters in KiSAO.

## S12 Funding

This work was supported by French National Research Agency award ANR-20-CE45-0001 to Paulevé; German Federal Ministry of Education and Research awards 031L0104A and 01ZX1916A to Weindl; German Research Foundation award 391070520 to Brusch; Human Frontier Science Program award LT000259/2019-L1 to Fröhlich; US National Cancer Institute award U54CA225088 to Fröhlich; National Institute of Allergy and Infectious Disease to Zhang; US National Institute of Bioengineering and Bioimaging awards P41EB023912 to Gennari, Karr, Moraru, Nickerson, and Sauro and R01EB014877 to Drawert and Petzold; US National Institute of General Medical Sciences awards R24GM137787 to Moraru and R35GM119771 to Karr; and Wellcome Trust award 212941 to Gleeson.

## S13 Acknowledgements

We thank Manuel Bernal Llinares (European Molecular Biology Laboratory - European Bioinformatics Institute) for assistance expanding the Identifiers.org database of namespaces for entities for biological research. We thank Jan Hasenauer (Universität Bonn) for help specifying the capabilities of the AMICI continuous kinetic simulation program. We thank James C. Schaff (Applied BioMath) for helping conceive BioSimulators. We thank Ciaran Welsh (University of Auckland) for help using libOmexMeta to validate the metadata inside simulation projects.

## Acronyms

**API** application programming interface

**BNGL** BioNetGen Language

**EDAM** thE Data And Methods ontology

**FBA** flux balance analysis

**FVA** Flux Variability Analysis

**GUI** graphical user interface

**HDF5** Hierarchical Data Format 5

**HPC** high-performance computing

**JSON** JavaScript Object Notation

**KiSAO** Kinetic Simulation Algorithm Ontology

**OCI** Open Container Initiative

**OMEX** Open Modeling EXchange

**ORCID** Open Researcher and Contributor ID

**PDF** Portable Document Format

**pFBA** Parsimonious FBA

**RDF** Resource Description Framework

**SBML** Systems Biology Markup Language

**SBML-comp** SBML hierarchical model composition package

**SBML-fbc** SBML flux balance constraints package

**SBML-qual** SBML qualitative models package

**SBO** Systems Biology Ontology

**SED-ML** Simulation Experiment Description Markup Language

| | |
|---|---|
| **SIF** | Singularity Image Format |
| **SIO** | Semanticscience Integrated Ontology |
| **URI** | Uniform Resource Identifier |
| **URL** | Uniform Resource Locator |
| **URN** | Uniform Resource Name |
| **XML** | Extensible Markup Language |
| **YAML** | Yet Another Markup Language |

# References


1. Fröhlich,F., Weindl,D., Schälte,Y., Pathirana,D., Paszkowski,Ł., Lines,G. T., Stapor,P. and Hasenauer,J. (04, 2021) AMICI: High-Performance Sensitivity Analysis for Large Ordinary Differential Equation Models. *Bioinformatics,* btab227.
2. Harris,L. A., Hogg,J. S., Tapia,J.-J., Sekar,J. A., Gupta,S., Korsunsky,I., Arora,A., Barua,D., Sheehan,R. P. and Faeder,J. R. (2016) BioNetGen 2.2: advances in rule-based modeling. *Bioinformatics,* **32**, 3366–3368.
3. Kolpakov,F., Akberdin,I., Kashapov,T., Kiselev,l., Kolmykov,S., Kondrakhin,Y., Kutumova,E., Mandrik,N., Pintus,S., Ryabova,A. et al. (2019) BioUML: an integrated environment for systems biology and collaborative analysis of biomedical data. *Nucleic Acids Res.,* **47**, W225–W233.
4. Müssel,C., Hopfensitz,M. and Kestler,H. A. (2010) BoolNet—an R package for generation, reconstruction and analysis of Boolean networks. *Bioinformatics,* **26**, 1378–1380.
5. Garg,A., Mohanram,K., Di Cara,A., De Micheli,G. and Xenarios,I. (2009) Modeling stochasticity and robustness in gene regulatory networks. *Bioinformatics,* **25**, i101–i109.
6. Stimberg,M., Brette,R. and Goodman,D. F. (2019) Brian 2, an intuitive and efficient neural simulator. *Elife,* **8**, e47314.
7. Olivier,B. G., Swat,M. J. and Moné,M. J. (2016) Modeling and simulation tools: from systems biology to systems medicine. *Syst. Med.,* pp. 441–463.
8. Klamt,S., Saez-Rodriguez,J. and Gilles,E. D. (2007) Structural and functional analysis of cellular networks with CellNetAnalyzer. *BMC Syst. Biol.,* **1**, 1–13.
9. Heirendt,L., Arreckx,S., Pfau,T., Mendoza,S. N., Richelle,A., Heinken,A., Haraldsdóttir,H. S., Wachowiak,J., Keating,S. M., Vlasov,V. et al. (2019) Creation and analysis of biochemical constraint-based models using the COBRA Toolbox v.3.0. *Nat. Protoc.,* **14**, 639–702.
10. Ebrahim,A., Lerman,J. A., Palsson,B. O. and Hyduke,D. R. (2013) COBRApy: constraints-based reconstruction and analysis for Python. *BMC Syst. Biol.,* **7**, 1–6.
11. Bergmann,F. T., Hoops,S., Klahn,B., Kummer,U., Mendes,P., Pahle,J. and Sahle,S. (2017) COPASI and its applications in biotechnology. *J. Biotechnol.,* **261**, 215–220.
12. Dhar,P. K., Takahashi,K., Nakayama,Y. and Tomita,M. (2006) E-Cell: Computer Simulation of the Cell. *Rev. Cell. Biol. Mol. Med.,*.

13. Varela,P. L., Ramos,C. V., Monteiro,P. T. and Chaouiya,C. (2018) EpiLog: A software for the logical modelling of epithelial dynamics. *F1000Res.,* **7**.

14. Rowe,E., Palsson,B. O. and King,Z. A. (2018) Escher-FBA: a web application for interactive flux balance analysis. *BMC Syst. Biol.,* **12**, 1–7.

15. Marmiesse,L., Peyraud,R. and Cottret,L. (2015) FlexFlux: combining metabolic flux and regulatory network analyses. *BMC Syst. Biol.,* **9**, 1–13.

16. Hari,A. and Lobo,D. (2020) Fluxer: a web application to compute, analyze and visualize genome-scale metabolic flux networks. *Nucleic Acids Res.,* **48**, W427–W435.

17. Batt,G., Besson,B., Ciron,P.-E., de Jong,H., Dumas,E., Geiselmann,J., Monte,R., Monteiro,P. T., Page,M., Rechenmann,F. et al. (2012) Genetic Network Analyzer: a tool for the qualitative modeling and simulation of bacterial regulatory networks. In *Bacterial Molecular Networks* pp. 439–462 Springer.

18. Garg,A., Di Cara,A., Xenarios,I., Mendoza,L. and De Micheli,G. (2008) Synchronous versus asynchronous modeling of gene regulatory networks. *Bioinformatics,* **24**, 1917–1925.

19. Drawert,B., Hellander,A., Bales,B., Banerjee,D., Bellesia,G., Daigle Jr,B. J., Douglas,G., Gu,M., Gupta,A., Hellander,S. et al. (2016) Stochastic simulation service: bridging the gap between the computational expert and the biologist. *PLoS Comput. Biol.,* **12**, e1005220.

20. Naldi,A., Hernandez,C., Abou-Jaoudé,W., Monteiro,P. T., Chaouiya,C. and Thieffry,D. (2018) Logical modeling and analysis of cellular regulatory networks with GINsim 3.0. *Front. Physiol.,* **9**, 646.

21. Watanabe,L., Nguyen,T., Zhang,M., Zundel,Z., Zhang,Z., Madsen,C., Roehner,N. and Myers,C. (2018) iBioSim 3: a tool for model-based genetic circuit design. *ACS Synth. Biol.,* **8**, 1560–1563.

22. Cannon,R. C., Gleeson,P., Crook,S., Ganapathy,G., Marin,B., Piasini,E. and Silver,R. A. (2014) LEMS: a language for expressing complex biological models in concise and hierarchical form and its use in underpinning NeuroML 2. *Front. Neuroinform.,* **8**, 79.

23. Butterworth,E., Jardine,B. E., Raymond,G. M., Neal,M. L. and Bassingthwaighte,J. B. (2013) JSim, an open-source modeling system for data analysis. *F1000Res.,* **2**.

24. Peters,M., Eicher,J. J., van Niekerk,D. D., Waltemath,D. and Snoep,J. L. (2017) The JWS Online simulation database. *Bioinformatics,* **33**, 1589–1590.

25. Boutillier,P., Maasha,M., Li,X., Medina-Abarca,H. F., Krivine,J., Feret,J., Cristescu,I., Forbes,A. G. and Fontana,W. (2018) The Kappa platform for rule-based modeling. *Bioinformatics,* **34**, i583–i592.

26. Roberts,E., Stone,J. E. and Luthey-Schulten,Z. (2013) Lattice Microbes: High-performance stochastic simulation method for the reaction-diffusion master equation. *J. Comput. Chem.,* **34**, 245–255.

27. Takizawa,H., Nakamura,K., Tabira,A., Chikahara,Y., Matsui,T., Hiroi,N. and Funahashi,A. (2013) LibSBMLSim: a reference implementation of fully functional SBML simulator. *Bioinformatics,* **29**, 1474–1476.

28. Stoll,G., Caron,B., Viara,E., Dugourd,A., Zinovyev,A., Naldi,A., Kroemer,G., Barillot,E. and Calzone,L. (2017) MaBoSS 2.0: an environment for stochastic Boolean modeling. *Bioinformatics,* **33**, 2226–2228.

29. Haiman,Z. B., Zielinski,D. C., Koike,Y., Yurkovich,J. T. and Palsson,B. O. (2021) MASSpy:

Building, simulating, and visualizing dynamic biological models in Python using mass action kinetics. *PLoS Comput. Biol.,* **17**, e1008208.

30. Stiles,J. R., Bartol,T. M. et al. (2001) Monte Carlo methods for simulating realistic synaptic microphysiology using MCell. *Comput. Neurosci.,* pp. 87–127.
31. Moretti,S., Martin,O., Van Du Tran,T., Bridge,A., Morgat,A. and Pagni,M. (2016) MetaNetX/MNXref–reconciliation of metabolites and biochemical reactions to bring together genome-scale metabolic networks. *Nucleic Acids Res.,* **44**, D523–D526.
32. Swat,M., Moodie,S., Wimalaratne,S., Kristensen,N., Lavielle,M., Mari,A., Magni,P., Smith,M., Bizzotto,R., Pasotti,L. et al. Pharmacometrics Markup Language (PharmML): opening new perspectives for model exchange in drug development. (2015).
33. Starruß,J., De Back,W., Brusch,L. and Deutsch,A. (2014) Morpheus: a user-friendly modeling environment for multiscale and multicellular systems biology. *Bioinformatics,* **30**, 1331–1332.
34. Dura-Bernal,S., Suter,B. A., Gleeson,P., Cantarelli,M., Quintana,A., Rodriguez,F., Kedziora,D. J., Chadderdon,G. L., Kerr,C. C., Neymotin,S. A. et al. (2019) NetPyNE, a tool for data-driven multiscale modeling of brain circuits. *Elife,* **8**, e44494.
35. Hines,M. L. and Carnevale,N. T. (1997) The NEURON simulation environment. *Neural Comput.,* **9**, 1179–1209.
36. Erdemir,A. (2016) Open Knee: open source modeling & simulation to enable scientific discovery and clinical care in knee biomechanics. *J. Knee Surg.,* **29**, 107.
37. Garny,A. and Hunter,P. J. (2015) OpenCOR: a modular and interoperable approach to computational biology. *Front. Physiol.,* **6**, 26.
38. Seth,A., Hicks,J. L., Uchida,T. K., Habib,A., Dembia,C. L., Dunne,J. J., Ong,C. F., DeMers,M. S., Rajagopal,A., Millard,M. et al. (2018) OpenSim: Simulating musculoskeletal dynamics and neuromuscular control to study human and animal movement. *PLoS Comput. Biol.,* **14**, e1006223.
39. Rocha,I., Maia,P., Evangelista,P., Vilaça,P., Soares,S., Pinto,J. P., Nielsen,J., Patil,K. R., Ferreira,E. C. and Rocha,M. (2010) OptFlux: an open-source software platform for in silico metabolic engineering. *BMC Syst. Biol.,* **4**, 1–12.
40. Lopez,C. F., Muhlich,J. L., Bachman,J. A. and Sorger,P. K. (2013) Programming biological models in Python using PySB. *Mol. Syst. Biol.,* **9**, 646.
41. Olivier,B. G., Rohwer,J. M. and Hofmeyr,J.-H. S. (2005) Modelling cellular systems with PySCeS. *Bioinformatics,* **21**, 560–561.
42. Agren,R., Liu,L., Shoaie,S., Vongsangnak,W., Nookaew,I. and Nielsen,J. (2013) The RAVEN toolbox and its use for generating a genome-scale metabolic model for Penicillium chrysogenum. *PLoS Comput. Biol.,* **9**, e1002980.
43. Bulović,A., Fischer,S., Dinh,M., Golib,F., Liebermeister,W., Poirier,C., Tournier,L., Klipp,E., Fromion,V. and Goelzer,A. (2019) Automated generation of bacterial resource allocation models. *Metab. Eng.,* **55**, 12–22.
44. Panchiwala,H., Shah,S., Planatscher,H., Zakharchuk,M., König,M. and Dräger,A. (2022) The Systems Biology Simulation Core Library. *Bioinformatics,* **38**, 864–865.
45. Angermann,B. R. and Meier-Schellersheim,M. (2019) Using Python for Spatially Resolved Modeling with Simmune. In *Modeling Biomolecular Site Dynamics* pp. 161–177 Springer.
46. Updegrove,A., Wilson,N. M., Merkow,J., Lan,H., Marsden,A. L. and Shadden,S. C. (2017)

SimVascular: an open source pipeline for cardiovascular simulation. *Annal. Biomed. Eng.,* **45**, 525–541.

47. Andrews,S. S. (2017) Smoldyn: particle-based simulation with rule-based modeling, improved molecular interaction and a library interface. *Bioinformatics,* **33**, 710–717.

48. Choi,K., Medley,J. K., König,M., Stocking,K., Smith,L. P., Gu,S. and Sauro,H. M. (2018) tellurium: an extensible python-based modeling environment for systems and synthetic biology. *Biosystems,* **171**, 74–79.

49. Helikar,T., Kowal,B. and Rogers,J. (2013) A cell simulator platform: the Cell Collective. *Clin. Pharmacol. Ther.,* **93**, 393–395.

50. Moraru,I. I., Schaff,J. C., Slepchenko,B. M., Blinov,M., Morgan,F., Lakshminarayana,A., Gao,F., Li,Y. and Loew,L. M. (2008) Virtual Cell modelling and simulation software environment. *IET Syst. Biol.,* **2**, 352–362.

51. Sekiguchi,T., Hamada,H. and Okamoto,M. (2019) WinBEST-KIT: Biochemical reaction simulator that can define and customize algebraic equations and events as GUI components. *J. Bioinform. Comput. Biol.,* **17**, 1950036.

52. Ermentrout,B. (2012) XPPAUT. In *Computational Systems Neurobiology* pp. 519–531 Springer.

53. Faeder,J. R., Blinov,M. L. and Hlavacek,W. S. (2009) Rule-based modeling of biochemical systems with BioNetGen. In *Systems Biology* pp. 113–167 Springer.

54. Clerx,M., Cooling,M. T., Cooper,J., Garny,A., Moyle,K., Nickerson,D. P., Nielsen,P. M. and Sorby,H. (2020) CellML 2.0. *J. Integr. Bioinform.,* **17**, 20200021.

55. Gleeson,P., Crook,S., Cannon,R. C., Hines,M. L., Billings,G. O., Farinella,M., Morse,T. M., Davison,A. P., Ray,S., Bhalla,U. S. et al. (2010) NeuroML: a language for describing data driven models of neurons and networks with a high degree of biological detail. *PLoS Comput. Biol.,* **6**, e1000815.

56. Keating,S. M., Waltemath,D., König,M., Zhang,F., Dräger,A., Chaouiya,C., Bergmann,F. T., Finney,A., Gillespie,C. S., Helikar,T. et al. (2020) SBML Level 3: an extensible format for the exchange and reuse of biological models. *Mol. Syst. Biol.,* **16**, e9110.

57. Olivier,B. G. and Bergmann,F. T. (2018) SBML level 3 package: flux balance constraints version 2. *J. Integr. Bioinform.,* **15**, 20170082.

58. Smith,L. and Hucka,M. (2010) SBML Level 3 hierarchical model composition. *Nat. Preced.,* pp. 1–1.

59. Zhang,F. and Meier-Schellersheim,M. (2018) SBML level 3 package: multistate, multicomponent and multicompartment species, version 1, release 1. *J. Integr. Bioinform.,* **15**.

60. Chaouiya,C., Bérenguier,D., Keating,S. M., Naldi,A., Van Iersel,M. P., Rodriguez,N., Dräger,A., Büchel,F., Cokelaer,T., Kowal,B. et al. (2013) SBML qualitative models: a model representation format and infrastructure to foster interactions between qualitative modelling formalisms and tools. *BMC Syst. Biol.,* **7**, 1–15.

61. Hairer,E., Nørsett,S. and Wanner,G. (2008) Solving Ordinary Differential Equations I: Nonstiff Problems, Springer Series in Computational MathematicsSpringer Berlin Heidelberg, .

62. Hairer,E., Nørsett,S. P. and Wanner,G. (1993) Solving ordinary differential equations I: Nonstiff problems, Springer Verlag, Berlin, 2nd edition.

63. Cao,Y., Gillespie,D. T. and Petzold,L. R. (2007) Adaptive explicit-implicit tau-leaping method

with automatic tau selection. *J. Chem. Phys.,* **126**, 224101.

64. Schwab,J. D., Kühlwein,S. D., Ikonomi,N., Kühl,M. and Kestler,H. A. (2020) Concepts in Boolean network modeling: What do they all mean?. *Comput. Struct. Biotechnol. J.,* **18**, 571–582.
65. Curtiss,C. F. and Hirschfelder,J. O. (1952) Integration of stiff equations. *Proc. Natl. Acad. Sci. U. S. A.,* **38**, 235.
66. Smoluchowski,M. V. (1917) Versuch Einer Mathematischen Theorie der Koagulationskinetik kolloider Lösungen. *Zeitschrift Physik. Chemie.,* **XCII**, 129–168.
67. Cash,J. R. and Karp,A. H. (sep, 1990) A Variable Order Runge-Kutta Method for Initial Value Problems with Rapidly Varying Right-Hand Sides. *ACM Trans. Math. Softw.,* **16**, 201–222.
68. Crank,J. and Nicolson,P. (1947) A practical method for numerical evaluation of solutions of partial differential equations of the heat-conduction type. In *Mathematical Proceedings of the Cambridge Philosophical Society* Cambridge University Press Vol. 43, pp. 50–67.
69. Cohen,S. D., Hindmarsh,A. C. and Dubois,P. F. (1996) CVODE, a stiff/nonstiff ODE solver in C. *Comput. Phys.,* **10**, 138–143.
70. Serban,R. and Hindmarsh,A. C. (2005) CVODES: the sensitivity-enabled ODE solver in SUNDIALS. In *Proc. Comput. Inform. Eng. Conf.* Vol. 47438, pp. 257–269.
71. Dormand,J. R. and Prince,P. J. (1980) A family of embedded Runge-Kutta formulae. *J. Comput. Appl. Math.,* **6**, 19–26.
72. Griffiths,D. F. and Higham,D. J. (2010) Numerical Methods for Ordinary Differential Equations: Initial Value Problems, Springer, London, .
73. Yaakub,A. and Evans,D. J. (1999) A fourth order Runge-Kutta RK (4, 4) method with error control. *Int. J. Comput. Math.,* **71**, 383–411.
74. Fehlberg,E. (1969) Low-order classical Runge-Kutta formulas with stepsize control and their application to some heat transfer problems, Vol. 315, National Aeronautics and Space Administration, .
75. Orth,J. D., Thiele,I. and Palsson,B. Ø. (2010) What is flux balance analysis?. *Nat. Biotechnol.,* **28**, 245–248.
76. Mahadevan,R. and Schilling,C. (2003) The effects of alternate optimal solutions in constraint-based genome-scale metabolic models. *Metabolic Eng.,* **5**, 264–276.
77. Eymard,R., Gallouët,T. and Herbin,R. (2000) Finite volume methods. *Handbook Numer. Anal.,* **7**, 713–1018.
78. Smallbone,K. and Simeonidis,E. (2009) Flux balance analysis: a geometric perspective. *J. Theor. Biol.,* **258**, 311–315.
79. Gibson,M. A. and Bruck,J. (2000) Efficient exact stochastic simulation of chemical systems with many species and many channels. *J. Phys. Chem. A,* **104**, 1876–1889.
80. Gillespie,D. T. (1977) Exact stochastic simulation of coupled chemical reactions. *J. Phys. Chem.,* **81**, 2340–2361.
81. Salis,H. and Kaznessis,Y. (2005) Accurate hybrid stochastic simulation of a system of coupled chemical or biochemical reactions. *J. Chem. Phys.,* **122**, 054103.
82. Pahle,J. Eine Hybridmethode zur Simulation biochemischer Prozesse PhD thesis Universitat Karlsruhe (2002).

83. Sahle,S., Gauges,R., Pahle,J., Simus,N., Kummer,U., Hoops,S., Lee,C., Singhal,M., Xu,L. and Mendes,P. (2006) Simulation of biochemical networks using COPASI-a complex pathway simulator. In *Proc. Winter Simul. Conf.* IEEE pp. 1698–1706.

84. Hindmarsh,A. C., Brown,P. N., Grant,K. E., Lee,S. L., Serban,R., Shumaker,D. E. and Woodward,C. S. (2005) SUNDIALS: Suite of nonlinear and differential/algebraic equation solvers. *ACM Trans. Math. Softw.,* **31**, 363–396.

85. Klarner,H., Bockmayr,A. and Siebert,H. (2015) Computing maximal and minimal trap spaces of Boolean networks. *Nat. Comput.,* **14**, 535–544.

86. Hindmarsh,A. C. and Petzold,L. R. (Sep, 2005) LSODA, Ordinary Differential Equation Solver for Stiff or Non-Stiff System, International Atomic Energy Agency, .

87. Hoffman,J. D. and Frankel,S. (2001) Numerical Methods for Engineers and Scientists, Taylor & Francis, 2nd edition.

88. Naldi,A., Thieffry,D. and Chaouiya,C. (2007) Decision diagrams for the representation and analysis of logical models of genetic networks. In *International Conference on Computational Methods in Systems Biology* Springer pp. 233–247.

89. Sneddon,M. W., Faeder,J. R. and Emonet,T. (2011) Efficient modeling, simulation and coarse-graining of biological complexity with NFsim. *Nat. Methods,* **8**, 177–183.

90. Nowak,U. and Weimann,L. (1992) A Family of Newton Codes for Systems of Highly Nonlinear Equations, Konrad-Zuse-Zentrum für Informationstechnik Berlin, .

91. Deuflhard,P. (2011) Newton methods for nonlinear problems: affine invariance and adaptive algorithms, Vol. 35, Springer Science & Business Media, .

92. Press,W. H., Teukolsky,S. A., Vetterling,W. T. and Flannery,B. P. (1992) Numerical Recipes in C (2nd Ed.): The Art of Scientific Computing, Cambridge University Press, USA.

93. Lewis,N. E., Hixson,K. K., Conrad,T. M., Lerman,J. A., Charusanti,P., Polpitiya,A. D., Adkins,J. N., Schramm,G., Purvine,S. O., Lopez-Ferrer,D. et al. (2010) Omic data from evolved E. coli are consistent with computed optimal growth from genome-scale models. *Mol. Syst. Biol.,* **6**, 390.

94. Schuetz,R., Kuepfer,L. and Sauer,U. (2007) Systematic evaluation of objective functions for predicting intracellular fluxes in Escherichia coli. *Mol. Syst. Biol.,* **3**, 119.

95. Harris,L. A. and Clancy,P. (2006) A "partitioned leaping" approach for multiscale modeling of chemical reaction dynamics. *J. Chem. Phys.,* **125**, 144107.

96. Rentrop,P. (1985) Partitioned Runge-Kutta methods with stiffness detection and stepsize control. *Numer. Math.,* **47**, 545–564.

97. Ding,X. and Tan,J. (2009) Implicit Runge-Kutta methods based on Radau quadrature formula. *Int. J. Comput. Math.,* **86**, 1394–1404.

98. Goelzer,A. and Fromion,V. (2011) Bacterial growth rate reflects a bottleneck in resource allocation. *Biochim. Biophys. Acta,* **1810**, 978–988.

99. Rosenbrock,H. (1963) Some general implicit processes for the numerical solution of differential equations. *Comput. J.,* **5**, 329–330.

100. Rößler,A. (2009) Second order Runge-Kutta methods for Itô stochastic differential equations. *SIAM J. Numer. Anal.,* **47**, 1713–1738.

101. Gillespie,D. T. (2000) The chemical Langevin equation. *J. Chem. Phys.,* **113**, 297–306.

102. Gillespie,D. T. (2001) Approximate accelerated stochastic simulation of chemically reacting systems. *J. Chem. Phys.,* **115**, 1716–1733.

103. Brown,P. N., Byrne,G. D. and Hindmarsh,A. C. (1989) VODE: A variable-coefficient ODE solver. *SIAM J. Sci. Stat. Comput.,* **10**, 1038–1051.

104. Folk,M., Heber,G., Koziol,Q., Pourmal,E. and Robinson,D. (2011) An overview of the HDF5 technology suite and its applications. In *Proc. EDBT/ICDT 2011 Workshop Array Databases* pp. 36–47.

105. Bergmann,F. T. et al. (2014) COMBINE archive and OMEX format: one file to share all information to reproduce a modeling project. *BMC Bioinformatics,* **15**, 1–9.

106. Waltemath,D., Adams,R., Bergmann,F. T., Hucka,M., Kolpakov,F., Miller,A. K., Moraru,I. I., Nickerson,D., Sahle,S., Snoep,J. L. et al. (2011) Reproducible computational biology experiments with SED-ML-the simulation experiment description markup language. *BMC Syst. Biol.,* **5**, 1–10.

107. Bergmann,F. T., Cooper,J., König,M., Moraru,I., Nickerson,D., Le Novère,N., Olivier,B. G., Sahle,S., Smith,L. and Waltemath,D. (2018) Simulation experiment description markup language (SED-ML) level 1 version 3 (L1V3). *J. Integr. Bioinform.,* **15**.

108. Smith,L. P., Bergmann,F. T., Garny,A., Helikar,T., Karr,J., Nickerson,D., Sauro,H., Waltemath,D. and König,M. (2021) The Simulation Experiment Description Markup Language (SED-ML): language specification for Level 1 Version 4. *J. Integr. Bioinform.,* **18**, 20210021.

109. Köhn,D., Le Novere,N. and Knüpfer,C. (2009) Beyond Structure: KiSAO and TEDDY–Two Ontologies Addressing Pragmatical and Dynamical Aspects of Computational Models in Systems Biology. *Nat. Preced.,* pp. 1–1.

110. Courtot,M., Juty,N., Knüpfer,C., Waltemath,D., Zhukova,A., Dräger,A., Dumontier,M., Finney,A., Golebiewski,M., Hastings,J. et al. (2011) Controlled vocabularies and semantics in systems biology. *Mol. Syst. Biol.,* **7**, 543.

111. Satyanarayan,A., Wongsuphasawat,K. and Heer,J. (2014) Declarative interaction design for data visualization. In *Proc. 27th Annu. ACM Symp. User Interface Soft. Technol.* pp. 669–678.

112. Ison,J., Kalaš,M., Jonassen,I., Bolser,D., Uludag,M., McWilliam,H., Malone,J., Lopez,R., Pettifer,S. and Rice,P. (2013) EDAM: an ontology of bioinformatics operations, types of data and identifiers, topics and formats. *Bioinformatics,* **29**, 1325–1332.

113. Black,M., Lamothe,L., Eldakroury,H., Kierkegaard,M., Priya,A., Machinda,A., Khanduja,U. S., Patoliya,D., Rathi,R., Nico,T. P. C., Umutesi,G., Blankenburg,C., Op,A., Chieke,P., Babatunde,O., Laurie,S., Neumann,S., Schwämmle,V., Kuzmin,I., Hunter,C., Karr,J., Ison,J., Gaignard,A., Brancotte,B., Ménager,H. and Kalaš,M. (2022) EDAM: the bioscientific data analysis ontology (update 2021). *F1000Res.,* **11**, 1.

114. Dumontier,M., Baker,C. J., Baran,J., Callahan,A., Chepelev,L., Cruz-Toledo,J., Del Rio,N. R., Duck,G., Furlong,L. I., Keath,N. et al. (2014) The Semanticscience Integrated Ontology (SIO) for biomedical research and knowledge discovery. *J. Biomed. Semant.,* **5**, 1–11.

115. Wickham,H. (2011) ggplot2. *Wiley Interdiscip. Rev. Comput. Stat.,* **3**, 180–185.

116. Satyanarayan,A., Moritz,D., Wongsuphasawat,K. and Heer,J. (2016) Vega-lite: A grammar of interactive graphics. *IEEE Trans. Vis. Comput. Graph.,* **23**, 341–350.

117. Kurtzer,G. M., Sochat,V. and Bauer,M. W. (2017) Singularity: Scientific containers for mo-

bility of compute. *PloS One,* **12**, e0177459.

118. da Veiga Leprevost,F., Grüning,B. A., Alves Aflitos,S., Röst,H. L., Uszkoreit,J., Barsnes,H., Vaudel,M., Moreno,P., Gatto,L., Weber,J. et al. (2017) BioContainers: an open-source and community-driven framework for software standardization. *Bioinformatics,* **33**, 2580–2582.
119. Agmon,E. and Spangler,R. K. (2020) A multi-scale approach to modeling E. coli chemotaxis. *Entropy,* **22**, 1101.
120. Agmon,E., Spangler,R. K., Skalnik,C. J., Poole,W., Peirce,S. M., Morrison,J. H. and Covert,M. W. (02, 2022) Vivarium: an interface and engine for integrative multiscale modeling in computational biology. *Bioinformatics,* btac049.
121. Haak,L. L., Fenner,M., Paglione,L., Pentz,E. and Ratner,H. (2012) ORCID: a system to uniquely identify researchers. *Learn. Publ.,* **25**, 259–264.
122. Welsh,C., Nickerson,D. P., Rampadarath,A., Neal,M. L., Sauro,H. M. and Gennari,J. H. (2021) libOmexMeta: enabling semantic annotation of models to support FAIR principles. *Bioinformatics,* **37**, 4898–4900.
123. Bernal-Llinares,M., Ferrer-Gómez,J., Juty,N., Goble,C., Wimalaratne,S. M. and Hermjakob,H. (2020) Identifiers.org: Compact Identifier services in the cloud. *Bioinformatics,* p. btaa864.
124. Naldi,A., Hernandez,C., Levy,N., Stoll,G., Monteiro,P. T., Chaouiya,C., Helikar,T., Zinovyev,A., Calzone,L., Cohen-Boulakia,S. et al. (2018) The CoLoMoTo interactive notebook: accessible and reproducible computational analyses for qualitative biological networks. *Front. Physiol.,* **9**, 680.
125. Singh,D. and Andrews,S. S. (2022) Python interfaces for the Smoldyn simulator. *Bioinformatics,* **38**, 291–293.
126. Zhukova,A., Adams,R., Laibe,C. and Le Novere,N. (2012) LibKiSAO: a Java library for Querying KiSAO. *BMC Res. Notes,* **5**, 1–7.
127. Shaikh,B., Marupilla,G., Wilson,M., Blinov,M. L., Moraru,I. I. and Karr,J. R. (2021) RunBioSimulations: an extensible web application that simulates a wide range of computational modeling frameworks, algorithms, and formats. *Nucleic Acids Res.,* **49**, W597–W602.
128. Hunter,J. D. (2007) Matplotlib: A 2D graphics environment. *IEEE Ann. Hist. Comput.,* **9**, 90–95.
129. Hagberg,A., Swart,P. and S Chult,D., Exploring network structure, dynamics, and function using NetworkX. Technical report, Los Alamos National Laboratory (2008).
130. Oliphant,T. E. (2006) A guide to NumPy, Vol. 1, Trelgol Publishing USA, .
131. Tiwari,K., Kananathan,S., Roberts,M. G., Meyer,J. P., Sharif Shohan,M. U., Xavier,A., Maire,M., Zyoud,A., Men,J., Ng,S. et al. (2021) Reproducibility in systems biology modelling. *Mol. Syst. Biol,* **17**, e9982.
132. Malik-Sheriff,R. S., Glont,M., Nguyen,T. V., Tiwari,K., Roberts,M. G., Xavier,A., Vu,M. T., Men,J., Maire,M., Kananathan,S. et al. (2020) BioModels—15 years of sharing computational models in life science. *Nucleic Acids Res.,* **48**, D407–D415.
133. Rew,R. and Davis,G. (1990) NetCDF: an interface for scientific data access. *IEEE Comput. Graph. Appl.,* **10**, 76–82.
134. Stanford,N. J., Scharm,M., Dobson,P. D., Golebiewski,M., Hucka,M., Kothamachu,V. B., Nickerson,D., Owen,S., Pahle,J., Wittig,U. et al. (2019) Data management in computational systems biology: exploring standards, tools, databases, and packaging best practices. In *Yeast*

*Systems Biology* pp. 285–314 Springer.

135. Dada,J. O., Spasić,I., Paton,N. W. and Mendes,P. (2010) SBRML: a markup language for associating systems biology data with models. *Bioinformatics,* **26**, 932–938.

136. Evans,T. W., Gillespie,C. S. and Wilkinson,D. J. (2008) The SBML discrete stochastic models test suite. *Bioinformatics,* **24**, 285–286.

137. Bergmann,F. T. and Sauro,H. M. (2008) Comparing simulation results of SBML capable simulators. *Bioinformatics,* **24**, 1963–1965.

138. Ison,J., Ienasescu,H., Chmura,P., Rydza,E., Ménager,H., Kalaš,M., Schwämmle,V., Grüning,B., Beard,N., Lopez,R. et al. (2019) The bio.tools registry of software tools and data resources for the life sciences. *Genome Biol.,* **20**, 1–4.

139. Schmiester,L., Schälte,Y., Bergmann,F. T., Camba,T., Dudkin,E., Egert,J., Fröhlich,F., Fuhrmann,L., Hauber,A. L., Kemmer,S. et al. (2021) PEtab—Interoperable specification of parameter estimation problems in systems biology. *PLoS Comput. Biol.,* **17**, e1008646.

140. Grethe,J., Bandrowski,A., Chiu,M., Gillespie,T., Go,J., Li,Y., Ozyurt,I. and Martone,M. (2016) SciCrunch: A Cooperative And Collaborative Data, Information, And Resource Discovery Portal For Scientific Communities. In *Proc. Neuroinform.*

141. Grüning,B. et al. (2018) Bioconda: sustainable and comprehensive software distribution for the life sciences. *Nat. Methods,* **15**, 475–476.

142. O'Connor,B. D., Yuen,D., Chung,V., Duncan,A. G., Liu,X. K., Patricia,J., Paten,B., Stein,L. and Ferretti,V. (2017) The Dockstore: enabling modular, community-focused sharing of Docker-based genomics tools and workflows. *F1000Res.,* **6**.

143. Blankenberg,D., Von Kuster,G., Bouvier,E., Baker,D., Afgan,E., Stoler,N., Taylor,J. and Nekrutenko,A. (2014) Dissemination of scientific software with Galaxy ToolShed. *Genome Biol.,* **15**, 1–3.

144. Arkin,A. P., Cottingham,R. W., Henry,C. S., Harris,N. L., Stevens,R. L., Maslov,S., Dehal,P., Ware,D., Perez,F., Canon,S. et al. (2018) KBase: the United States department of energy systems biology knowledgebase. *Nat. Biotechnol.,* **36**, 566–569.

145. Yamada,T. G., Ii,K., König,M., Feierabend,M., Dräger,A. and Funahashi,A. (2021) SBMLWebApp: Web-Based Simulation, Steady-State Analysis, and Parameter Estimation of Systems Biology Models. *Processes,* **9**, 1830.
